# Small RNA-Sequencing Links Physiological Changes and RdDM Process to Vegetative-to-Floral Transition in Apple

**DOI:** 10.3389/fpls.2017.00873

**Published:** 2017-05-29

**Authors:** Xinwei Guo, Zeyang Ma, Zhonghui Zhang, Lailiang Cheng, Xiuren Zhang, Tianhong Li

**Affiliations:** ^1^Department of Fruit Science, College of Horticulture, China Agricultural UniversityBeijing, China; ^2^Department of Biochemistry and Biophysics, Texas A&M UniversityCollege Station, TX, United States; ^3^Institute for Plant Genomics and Biotechnology, Texas A&M UniversityCollege Station, TX, United States; ^4^Guangdong Provincial Key Laboratory of Biotechnology for Plant Development, School of Life Science, South China Normal UniversityGuangzhou, China; ^5^Department of Horticulture, Cornell UniversityIthaca, NY, United States; ^6^Beijing Collaborative Innovation Center for Eco-Environmental Improvement with Forestry and Fruit TreesBeijing, China

**Keywords:** apple, vegetative and floral buds, floral transition, sRNA, miRNA, RdDM

## Abstract

Transition from vegetative to floral buds is a critical physiological change during flower induction that determines fruit productivity. Small non-coding RNAs (sRNAs) including microRNAs (miRNAs) and small interfering RNAs (siRNAs) are pivotal regulators of plant growth and development. Although the key role of sRNAs in flowering regulation has been well-described in Arabidopsis and some other annual plants, their relevance to vegetative-to-floral transition (hereafter, referred to floral transition) in perennial woody trees remains under defined. Here, we performed Illumina sequencing of sRNA libraries prepared from vegetative and floral bud during flower induction of the apple trees. A large number of sRNAs exemplified by 33 previously annotated miRNAs and six novel members display significant differential expression (DE) patterns. Notably, most of these DE-miRNAs in floral transition displayed opposite expression changes in reported phase transition in apple trees. Bioinformatics analysis suggests most of the DE-miRNAs targeted transcripts involved in *SQUAMOSA PROMOTER BINDING PROTEIN-LIKE* (*SPL*) gene regulation, stress responses, and auxin and gibberellin (GA) pathways, with further suggestion that there is an inherent link between physiological stress response and metabolism reprogramming during floral transition. We also observed significant changes in 24 nucleotide (nt) sRNAs that are hallmarks for RNA-dependent DNA methylation (RdDM) pathway, suggestive of the correlation between epigenetic modifications and the floral transition. The study not only provides new insight into our understanding of fundamental mechanism of poorly studied floral transition in apple and other woody plants, but also presents important sRNA resource for future in-depth research in the apple flowering physiology.

## Introduction

Apple (*Malus domestica* Borkh.) is a major deciduous fruit tree crop in the world. Similar to other woody plants, apple tree has a long juvenile phase (Zhang et al., 2007). After the phase transition to the adult stage, apple trees undergo annual reproductive growth cycle. During the annual flower induction period, the developmental transition from vegetative to reproductive growth takes place in the floral bud (Mimida et al., 2009). In apple trees, flower induction occurs in the preceding summer of spring bloom (Abbott, 1970). This time window is typically in July the Northern Hemisphere (Kurokura et al., 2013), and approximately 2 weeks prior to floral differentiation, depending on species and the geographic and climate conditions (Kotoda et al., 1999; Kotoda and Wada, 2005). As flower induction determines fruit development and fruit productivity in the next year to a large extent (Williamson and Darnell, 1999; Link, 2000), elucidating the mechanism regulating floral transition is critical for both apple breeding and cultivation (Foster et al., 2003; Bangerth, 2009).

Many internal and external factors regulate floral transition. In annual model plant Arabidopsis, numerous interwoven genetic pathways exemplified by vernalization, photoperiod, senescence, and phytohromone signaling could regulate flowering timing (Moon et al., 2005). On the molecular level, the above pathways can end at promotion of the flowering process by repression of flowering repressor *FLC* (*FLOWERING LOCUS C*) and *TFL1* (*TERMINAL FLOWER 1*), or by up-regulation of flowering activator *FT* (*FLOWERING LOCUS T*), *SOC1* (*SUPPERSSOR OF OVER EXPRESSION OF CONSTANS I*), *CO* (*COSTANS*), *AP1* (*APETALA1*), or *LFY* (*LEAFY*) (Teotia and Tang, 2015). Unlike annual plants that only flower one time during their life cycle, perennials live for many years and flower repeatedly (Albani et al., 2012). Moreover, for apple trees, the whole flowering process from floral bud initiation to blooming lasts for as long as 1 year. Thus, understanding the distinct mechanisms underline the floral transition in apple and other perennials is of high importance. Although some flowering-related genes have been cloned and analyzed in fruit crops, little is known about the sRNA's role in floral transition in apple and other fruit trees (Almada et al., 2009; Trankner et al., 2010; An et al., 2012; Lei et al., 2013; Porto et al., 2015; Wells et al., 2015; Ito et al., 2016).

miRNA plays pivotal roles in regulation of diverse biological processes including plant growth and development, flowering time, adaptation to the environment, and resistance to biotic and abiotic stress (Huijser and Schmid, 2011; Li et al., 2016). miRNAs are processed by Dicer-like machinery from primary miRNA precursors that contain a hairpin-like foldback, and then are incorporated into an Argonaute (AGO)-containing ribonucleoprotein complex to repress expression of target genes through cleavage of transcripts or translational repression in a sequence-specific manner (Zhang et al., 2015). In both annual model plant Arabidopsis and polycarpic perennial crops such as *Cardamine flexuosa*, miR156, and miR172 were first identified to regulate phase transition, the process that also represents the first time of floral transition during plant life circle. miR156 functions to extend juvenile phase and delay flowering, while miR172 leads to early flowering (Wu et al., 2009; Zhou C. M. et al., 2013). miR156 and miR172 could also regulate flowering time in response to vernalization through the opposite expression trends (Bergonzi et al., 2013). In Arabidopsis, miR156 targets a gene family of 11 *SQUAMOSA PROMOTER BINDING PROTEIN-LIKE* (*SPL*) transcription factors; whereas miR172 regulates six members of the *APETALA2* (*AP2*) transcription factors.

In apple, dozens of novel miRNAs or apple-specific miRNAs have been proposed through high-throughput sequencing (Xia et al., 2012; Ye et al., 2013; Xing et al., 2016). Meanwhile, more than 200 potential miRNA targets have been computationally predicted (Ye et al., 2013). Ectopic expression of apple miR156 h reduces expression levels of *AtSPL9* and *AtSPL15*, and delays the flowering time in transgenic Arabidopsis (Sun et al., 2013). On the other hand, constitutive expression of apple miR172 leads to earlier flowering in transgenic Arabidopsis (Zhao et al., 2015). Notably, numerous miRNAs related to the abscisic acid (ABA) and gibberellins (GA) pathways, flowering gene expression and floral bud formation have been reported to respond to shoot bending that promotes apple flower induction (Xing et al., 2016). To date, the relevance of miRNAs to developmental transition from vegetative to floral buds remains poorly described in apple trees.

Different from miRNAs, another group of sRNAs, namely, 24 nucleotide (nt) small interfering RNA (siRNA), function in nucleus to repress target loci through epigenetic silencing. Briefly, in model plant Arabidopsis, siRNAs are derived from endogenous loci and repetitive sequences, and are loaded into AGO4 and/or its genetic paralogs like AGO3/6/9 to target sequence-complementary loci to eventually trigger chromatin methylation through a group of DNA and histone methyltransfereases (Borges and Martienssen, 2015; Du et al., 2015). However, correlation between the 24-nt siRNAs and floral transition in plants remains under defined.

In this study, we aimed to investigate the potential connection of miRNAs and siRNAs in apple vegetative-to-floral bud transition. We identified differentially expressed miRNAs and siRNAs between vegetative and floral buds through small RNA sequencing data analysis. Bioinformatics analysis of the sRNAs sheds new light on our understanding of floral transition in woody plants, and provide a new idea to design strategies to accelerate apple-breeding process.

## Materials and methods

### Plant material and growth condition

Sixteen-year-old *Malus domestica* “Golden Delicious”/M.26 trees grown at an experimental orchard of Cornell University, New York, USA (40° 43′ N, 74° 0′ W) were used in this study. Lateral buds from extension shoots and terminal buds from non-fruiting spurs were taken during flower induction process in late July of 2015, and designated as vegetative bud (VB) and floral buds (FB), respectively. There were three biological replicates per bud type. For each replicate, the VB and the FB were collected from the same tree. Each sample contained 20 buds. The collected samples were immediately frozen in liquid nitrogen and stored at −80°C.

### sRNA extraction and library construction

For each sample, 100 mg of the ground powder was used for sRNA extraction as previously described (Wang C. et al., 2011). Six micro-grams (μg) of total RNA were spiked with 5′ ^32^P-labeled radioisotope-labeled 19–24 nt RNA oligos and then resolved in 15% urea-PAGE. sRNA library was constructed as previously described (Zhang et al., 2016). Briefly, sRNAs of 19–24 nt were gel-purified and ligated to a pair of adapters at the 5′ and 3′ ends by using T4 RNA ligase. sRNAs with adapters were transcribed into cDNA and amplified by Illumina sequencing-compatible primers. The final PCR products were sequenced at Texas A&M sequencing center. The sequences of 3′ and 5′ adapters, primers for amplification of the cDNA libraries are listed in Table S1.

### RNA blot analysis

sRNA blot assays were performed with aliquots of total RNAs used for sRNA-seq according to Zhang et al. (2016). Each lane contained 6 μg of total RNA. Blots were hybridized with ^32^P-radiolabeled oligo nucleotide probes complementary to related sRNAs. *U6* served as loading controls. sRNA blots were detected after exposure to a phosphor plate and quantified using the Quantity One Version 4.6.9 according to the manufacturer's instructions. Probes for RNA blot were listed in Table S1.

### Bioinformatic analysis

The sRNA reads from HiSeq 2000 were cleaned by passing the QC with the standard Illumina software first. After trimming adapters, sRNAs with lengths between 19- and 28-nt were selected and mapped using bowtie (version 1.1.2) to the *Malus* genomic sequences (*Malus domestica* Whole Genome v1.0 from http://www.rosaceae.org) with perfect genomic matches. The genomic and features of sRNAs were defined by the same version of genome annotation files. The previously annotated miRNAs were mapped to the reference genome *M. domestica* in miRase (release 21.0). sRNAs reads mapped to selected genomic features or miRNAs were count by BED Tools (v2.26) with 1 bp overlapping. All the loci with at least 1 readcount were retained, then edgeR (v3.3) were used for the differential expression (DE) analysis by normalization to total reads for each sample (Robinson et al., 2010). miRNAs with false discovery ration (FDR) < 0.05 were defined as DE-miRNAs.

Novel miRNA were first predicted by miRPlant V5 with cut off score over 0. Their secondary structures were predicted by PsRobot with the criteria of having large stem loop; mismatches in small RNA region between 0 and 7, and maximal precursor length of 200 nt (Wu et al., 2012; An et al., 2014). Then these candidates were examined individually according to the criteria from Meyers et al. (2008): (1) The miRNA and miRNA^*^ are derived from opposite stem-arms such that they form a duplex with two nucleotide, 3′-end overhangs; (2) base-pairing between the miRNA and the other arm of the hairpin, which includes the miRNA^*^, is extensive such that there are typically four or fewer mismatched miRNA bases; (3) asymmetric bulges are minimal in size (one or two bases) and frequency (typically one or less), especially within the miRNA/miRNA^*^ duplex. Only miRNAs that meet at least two criteria and showed consistent hairpin shaped secondary structures predicted by PsRobot (Wu et al., 2012), mfold (http://mfold.rna.albany.edu/?q= mfold), and miRPlant V5 were selected as final candidates.

miRNA targets were also predicted by PsRobot (Wu et al., 2012). Our criteria included: (1) Penalty score threshold = 2 (the penalty score of each candidate alignment is obtained by subtracting the actual alignment score from the ideal perfect global pairing score); (2) Maximal number of permitted mismatch = 1; (3) Position of mature miRNA sequence after which mismatch permitted is 17th. Gene ontology (GO) analysis (http://bioinfo.cau.edu.cn/agriGO/) was performed to classify the predicted target genes.

## Results

### Cloning of sRNAs from vegetative and floral bud in apple trees

**Figure 1:**
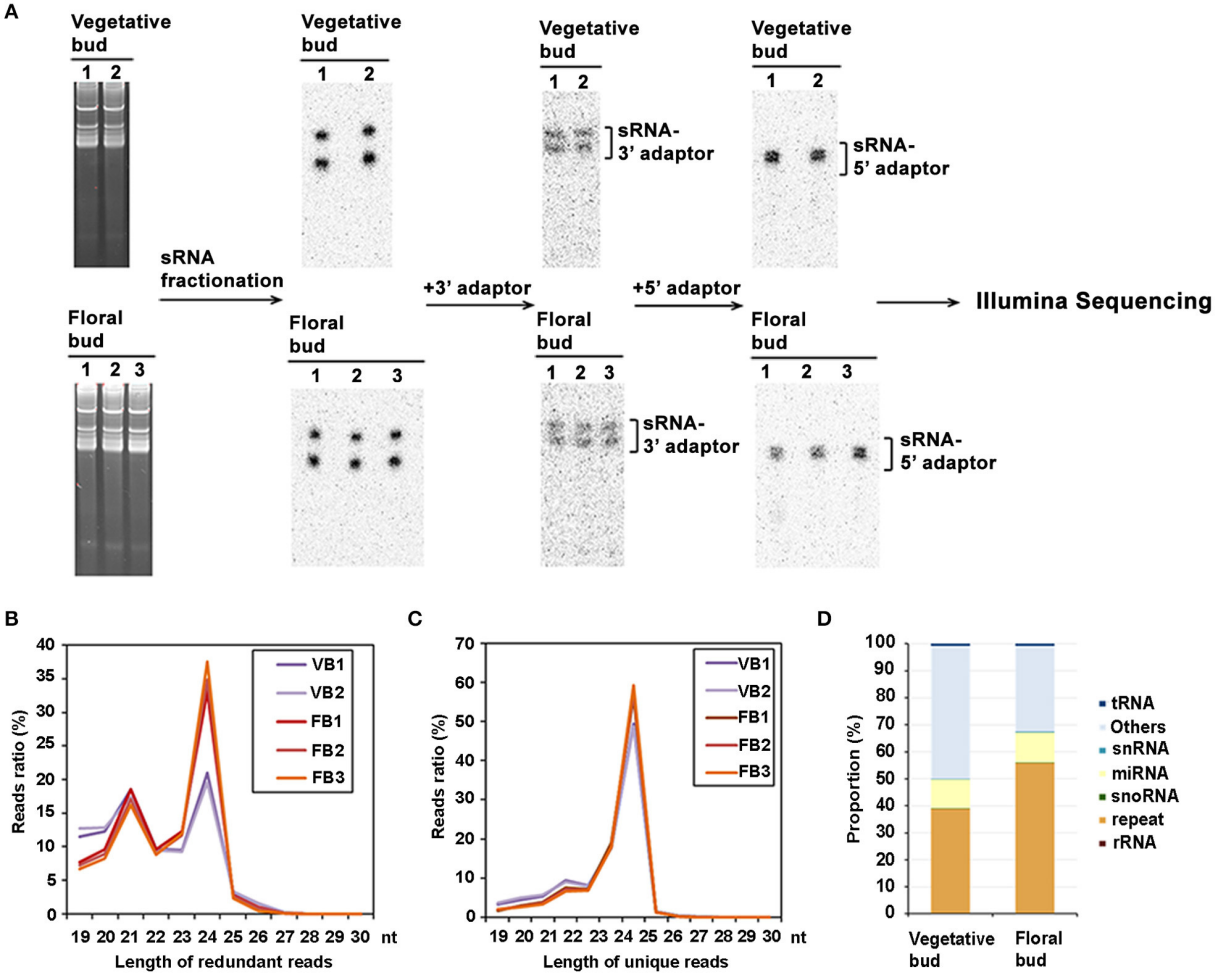
**Cloning of sRNAs from vegetative and floral bud in *Malus domestica*. (A)** RNA extraction and cloning of sRNA from vegetative and floral bud. **(B,C)** Size distribution of redundant **(B)** and unique **(C)** reads of sRNAs from vegetative and floral bud. **(D)** Genomic features of sRNA prepared from vegetative and floral bud.

To identify sRNAs potentially involved in apple flower induction, libraries of sRNAs prepared from vegetative and floral bud from *M. domestic* “Golden delicious” were constructed following the steps shown in Figure 1A. Two repeats for the vegetative bud (VB1 and VB2) and three repeats for the floral bud (FB1, FB2, and FB3) were finally obtained respectively. 5.2–9.1 million of reads were generated from each sample. Approximately 87–90% of the reads were perfectly mapped to the apple genome and included in further analysis (Table 1). In lines with previous reports, the reads of sRNAs were dominated by 21-nt and 24-nt long species, with the population of 24-nt sRNAs much larger than the 21-nt ones for both vegetative and floral bud (Figures 1B,C, Tables S2, S3). Related data have been deposited in Gene Expression Omnibus (GEO; http://www.ncbi.nlm.nih.gov/geo/query/acc.cgi?acc=GSE97777).

**Table 1:** **Statistics of raw and clean reads of small RNAs isolated from *Malus domestica***.

**Sample**	**Raw reads**	**Mapped reads**	**Mapped percentage**	**Unmapped reads**	**Unmapped percentage**
VB1	6018158	5339332	88.72	678826	11.28
VB2	5268927	4718634	89.56	550293	10.44
FB1	9078685	7910981	87.14	1167704	12.86
FB2	7470033	6518364	87.26	951669	12.74
FB3	7565678	6599438	87.23	966240	12.77

We then studied genomic features of the sRNAs. A substantial number of sRNAs (approximately 39–56%), were derived from repeat elements for all samples. Other species of RNAs detected included miRNAs (9.6–11.2%), small nucleolar RNA (snoRNAs) (0.1–0.2%), and small nuclear RNA (snRNAs) (0.2–0.3%). Notably, additional and in a large number, sRNAs were mapped to unknown regions for both the vegetative and floral buds (Figure 1D).

### Differentially expressed miRNAs in floral transition of apple trees

**Figure 2:**
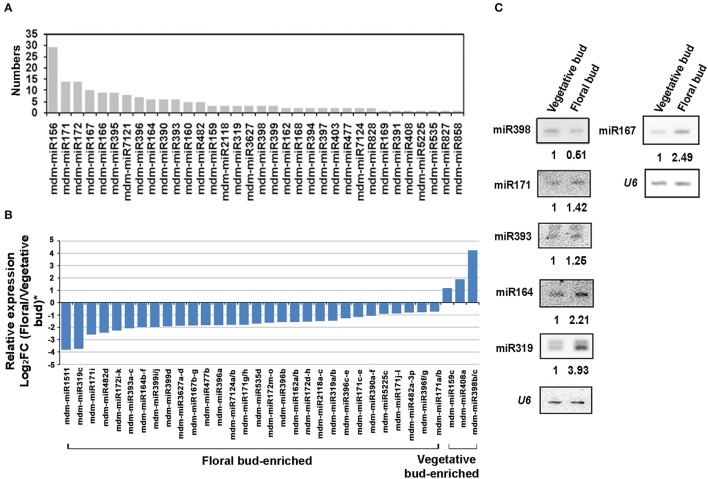
**Differentially expressed miRNAs during floral transition of *Malus domestica*. (A)** Numbers of known miRNA family members identified in apple vegetative and floral bud. **(B)** Expression pattern of differentially expressed known miRNAs (DE known miRNAs) between vegetative and floral bud. Expression level was set as Log_2_Fold Change (FC, floral bud/vegetative bud). **(C)** RNA gel blot for some DE known miRNAs. *U6* served as a loading control. ^*^All the values were given as Log_2_CPM (Counts per million).

The sRNAs in vegetative and floral bud libraries were queried using the known mature miRNAs of *M. domestica* in the miRBase 21.0 (http://www.mirbase.org/) database. The expression levels of these miRNAs were determined by normalizing their reads to the snoRNA reads, followed by comparison of their ratios in floral and vegetative bud (Log_2_FC-value). As such, 170 conserved miRNAs which belong to 34 families were finally identified (Table S4). Among these families, major *malus* miRNAs, including miRNA156 (9 members), miRNA171 (14), miRNA172 (14), miRNA167 (10), and miRNA395 (9), were detected (Figure 2A).

A number of 33 differentially expressed miRNAs (DE-miRNAs) (FDR < 0.05) between vegetative and floral bud were identified (Figure 2B). The expression of some DE-miRNAs was validated by RNA blot analysis (Figure 2C). Approximate 91% of the 33 DE-miRNAs showed enhanced expression in floral bud, whereas only levels of mdm-miR398b/c, mdm-miR408a, and mdm-miR159c enriched in vegetative bud. The flowering regulators miR156 expressed stronger in the vegetative buds, while miR172 was abundant in the floral buds (Table S4). Notably, mdm-miR398b/c, instead of miR156 or miR172, represents the most significant difference among all the DE-miRNAs (Figure 2B, Table S4).

### miRNAs in floral transition of apple trees

Next we predicted the potential targets for DE-miRNAs and the potential gene networks by using PsRobot (Wu et al., 2012; http://omicslab.genetics.ac.cn) as described in Methods. Under those scenarios, bioinformatics analysis revealed numerous targets for all miRNAs (Table 2). Notably, GO ontology analysis revealed that the potential targets of DE-miRNAs were mainly related to *SPL* gene regulation, stress response, and hormone pathways, providing three important clues to underline the mechanism of floral transition (Table 2, Table S5).

**Table 2:** **GO analysis of potential targets of differentially expressed known miRNAs during floral transition of *Malus domestica***.

**Name**	**Target protein**	**Protein ID**	**GO terms**	**GO ID**	**Function in plants**
**FLORAL TRANSITION AND PHASE TRANSITION RELATED**					
miR156	Squamosa promoter-binding protein-like (SPL; Sun et al., 2013)^**^	MDP0000155354 MDP0000158607 MDP0000146640 MDP0000297978 MDP0000263766	DNA binding	GO:0003677	Vegetative-to-floral phase transition Stress response Anthocyanin pathway
miR172	AP2 (Zhao et al., 2015)^**^	MDP0000281079 MDP0000130802 MDP0000137561 MDP0000204900 MDP0000163645 MDP0000200319	Transcription factor	GO:0003700	Vegetative-to-floral phase transition Stress response
**AUXIN PATHWAY**					
miR393	RNA polymerase III subunit RPC82 family protein^*^	MDP0000249678	DNA-directed RNA polymerase	GO:0003899	Auxin pathway
	F-box/RNI-like superfamily protein (TIR1)^*^	MDP0000498419	DNA binding	GO:0003677	
	DNA-directed RNA polymerase	MDP0000313025	Protein binding	GO:0005515	
miR167	Auxin response factor 6 (ARF6)^*^	MDP0000550049	Protein dimerization activity DNA binding	GO:0046983 GO:0003677	Auxin pathway Light inducible
miR390	Auxin response factor (At: ARF2/3/4)	MDP0000568581 MDP0000475144 MDP0000284274 MDP0000284274 MDP0000379904	Protein tyrosine kinase activity Protein binding	GO:0004713 GO:0005515	Auxin pathway
miR164	NAM/ATAF/CUC (NAC) domain containing protein1 (NAC1)^*^	MDP0000911724 MDP0000121265	DNA binding	GO:0003677	Multiple development stage Auxin pathway
**STRESS RESPONSE**					
miR398	DC1 domain-containing protein^*^	MDP0000152817	Protein-disulfide reductase Nucleotide binding	GO:0047134 GO:0000166	Oxidative stress Nutritional stress
	GroES-like zinc-binding dehydrogenase family protein^*^	MDP0000193167			
	Ctr copper transporter family^*^	MDP0000530255	Oxidoreductase activity	GO:0016491	Photosynthesis
miR408	Plantacyanin (ARPN)^*^	MDP0000124552 MDP0000150953	Copper ion binding	GO:0005507	Oxidative stress Nutritional stress Anthocyanin pathway
miR399	Inorganic phosphate transmembrane transporter [At: PHOSPHATE2 (PHO2)]	MDP0000166425	Transmembrane transport	GO:0055085	Nutritional stress
miR482	Plant invertase/pectinmethylesterase inhibitor superfamily^*^	MDP0000296741	Cell wall Pectinesterase	GO:0005618 GO:0030599	Defense response Conserved in trees
	Protein serine/threonine kinase	MDP0000305229	Protein binding	GO:0005515	
miR2118	Unknown (At: TIP-NBS-LRR)	MDP0000184039	Protein serine/threonine kinase		Stress response phasiRNA biogenesis
miR159	Transcription factor	MDP0000233948 MDP0000574158	Transcription factor Nucleus	GO:0003700 GO:0005634	Pollen tube growth Abiotic stress Defining plant morphology
	MYB domain protein 65 (MYB65)^*^	MDP0000321057			
miR1511	Ca2+ binding	MDP0000283945	Peptidyl-prolyl cis-trans isomerase activity	GO:0003755	Abiotic tress response
	Isomerase	MDP0000293965			
miR319	TEOSINTEBRANCHED1/CYCLOIDEA/ PRO-LIFERATING CELL FACTOR transcription factor4 (EE35,TCP4)^*^	MDP0000916623	Regulation of development	GO:0045962	Cell proliferation Abiotic stress response
miR171	DNA binding [At: Scarecrow-like proteins (SCL)]	MDP0000288614	DNA binding	GO:0006298 GO:0005524 GO:0030983	Plant development Light inducible
miR396	Transcription regulator [At; Growth-regulating factor (GRF)]	MDP0000194223	Transcription activator activity	GO:0016563	Cell proliferation Secondary metabolism
**TREE SPECIFIC**					
miR3627	Integral to membrane cellular component	MDP0000941000	Unknown	Unanootated	Conserved in trees
	AT hook motif DNA-bindingprotein^*^	MDP0000237744			
miR477	Unknown	MDP0000232264	Unknown	Unannotated	Conserved in trees
	Transcription factor	MDP0000431628			
miR7124	WD40/YVTN repeat-like domain	MDP0000162030 MDP0000296050	Transmembrane transport	GO:0055085	Only found in apple
	P-loop containing nucleosidetriphosphate hydrolases superfamily protein^*^	MDP0000319328	Microtubule-based movement	GO:0007018	
**OTHERS**					
miR162	Leucine zipper EF-hand containing transmembrane protein 1 (LETM1-LIKE protein)^*^ (At:DCL1)	MDP0000187512	Developmental growth	GO:0048589	miRNA processing
miR5225	RNA-directed RNA polymerase	MDP0000303619	RNA-directed RNA polymerase oxidation reduction	GO:0003968 GO:0055114	Unknown
miR535	Transferase activity (At: SPLs)	MDP0000177623	Biosynthetic process	GO:0009058	Plant development
		MDP0000173587	Protein binding	GO:0005515	

** *Experimental evidence in apple that involved the function analysis by transgene*.

* *Degradome sequencing data in apple from Xing et al. (2014) and Xing et al. (2016)*.

Several DE-miRNAs were shown to correlate with *SPL* genes, such as the vegetative bud-enriched miR156, miR159, miR398, and miR408. They were all reported to either target *SPLs* or involve in the *SPL* regulation (Wu et al., 2009; Yamasaki et al., 2009; Zhang et al., 2014). Besides, miR159 involves in GA pathway during flower development (Reyes and Chua, 2007).

Many DE-miRNAs in our results were reported to be stress responsive, such as the floral bud-enriched ones including miR159, miR171, miR319, miR396, miR399, miR482, miR1511, and miR2118 (Table 2). They were reported to involve in various abiotic (e.g., copper, drought, or ABA) and biotic stress resistance (e.g., fungal pathogen; Abdel-Ghany and Pilon, 2008; Jia et al., 2009; Pantaleo et al., 2010; Zhai et al., 2011; Zhou M. et al., 2013; Ma Z. et al., 2014).

Auxin is a phytohromone that play a critical role in plant growth and development including the flowering process. Of the DE-miRNAs detected, miR164 targets *NAC* (*NAM, ATAF, CUC*) genes, miR167 targets *AUXIN RESPONSIVE FACTOR6/8* (*ARF*6/8), miR390 targets trans-acting small interfering RNA3 (TAS3) transcripts to produce ta-siRNAs, which in turn regulates plant development by repressing *ARF2/3/4*, and miR393 targets *TIR1* genes. All the above miRNAs were up-regulated in floral bud, indicating the potential changes on auxin response during the floral transition.

In addition, some of the DE-miRNAs have been reported to regulate floral development in some other plants. For instance, miR396, which was expressed higher in floral bud, was reported to target *GROWTH REGULATING FACTORS* (*GRFs*) and to regulate cell proliferation and function in floral organ specification in *Populus* (Yang C. Y. et al., 2015). Last but not least, some DE-miRNAs, exemplified by miR1522, have been also recovered but their functions appear to be elusive at this stage. Some others such as miR3627, miR477, and miR7124 were specific to trees or apple according to miRBase 21.0 but their roles remain to be explored yet.

### Re-annotation of recently reported new miRNAs from apple tree

Recent effort of sRNA-seq in the different materials from apple has recovered 349 novel miRNAs (Ma C. et al., 2014; Xing et al., 2014; Kaja et al., 2015). We mined the public database and compared them with ours. Among them, 172 novel miRNAs were predicted to involve in phase transition in apple trees (Xing et al., 2014). To our surprise, only 17 novel miRNAs could be detected in our materials (Table S6). Among them, 10 showed opposite patterns whereas the rest displayed consistent changes between our results and previous data (Figure S1A). Based on FDR-values, only four novel miRNAs showed significant changes in our data (Table S6). Specifically, three novel miRNAs displayed stronger expression in floral bud (which are designated as Xing-novel-mir334/276/4 here), whereas one novel miRNA had stronger expression in vegetative bud (designated as Xing-novel-mir262).

By sharp contrast, we were unable to detect the remaining tentative novel miRNAs reported (Xing et al., 2014). These sRNAs either had no hits in our database or numbers of the reads were extremely low, thus we considered these sRNAs might be siRNAs, rather *bona fide* miRNAs. Similarly, we failed to detect all novel miRNAs reported by Kaja et al. (2015) and Ma C. et al. (2014) in our materials. As we could not access the sequence, structure information, and reads mapping of precursors for the tentative miRNAs, we could not evaluate the viability of the reported novel miRNA candidates.

Then we did target prediction and GO analysis of the 17 novel miRNAs overlapped between our sRNA-seq and the one reported by Xing et al. (2014). For the novel miRNAs with a consistent pattern between our studies, their targets were mainly related to the cell component, catalytic, metabolic process (Figure S1B). While for the opposite part, the targets were related to stimulus response, signaling process and biological regulation, which might represents some of the differences between buds and leaves (Figure S1C, Table S7).

### Six differentially expressed novel miRNAs in floral transition of apple trees

We next aimed at identifying novel miRNAs that are potentially engaged in floral transition in apple tree. To this end, we mined the remaining unannotated reads that could be mapped to the *M. domestica* genomic exon antisense strand, intron and intergenic regions and conducted bioinformatics predict for potential novel miRNAs. Based on the standards in Methods, our initial screening recovered 425 candidates (Figure S2).

**Figure 3:**
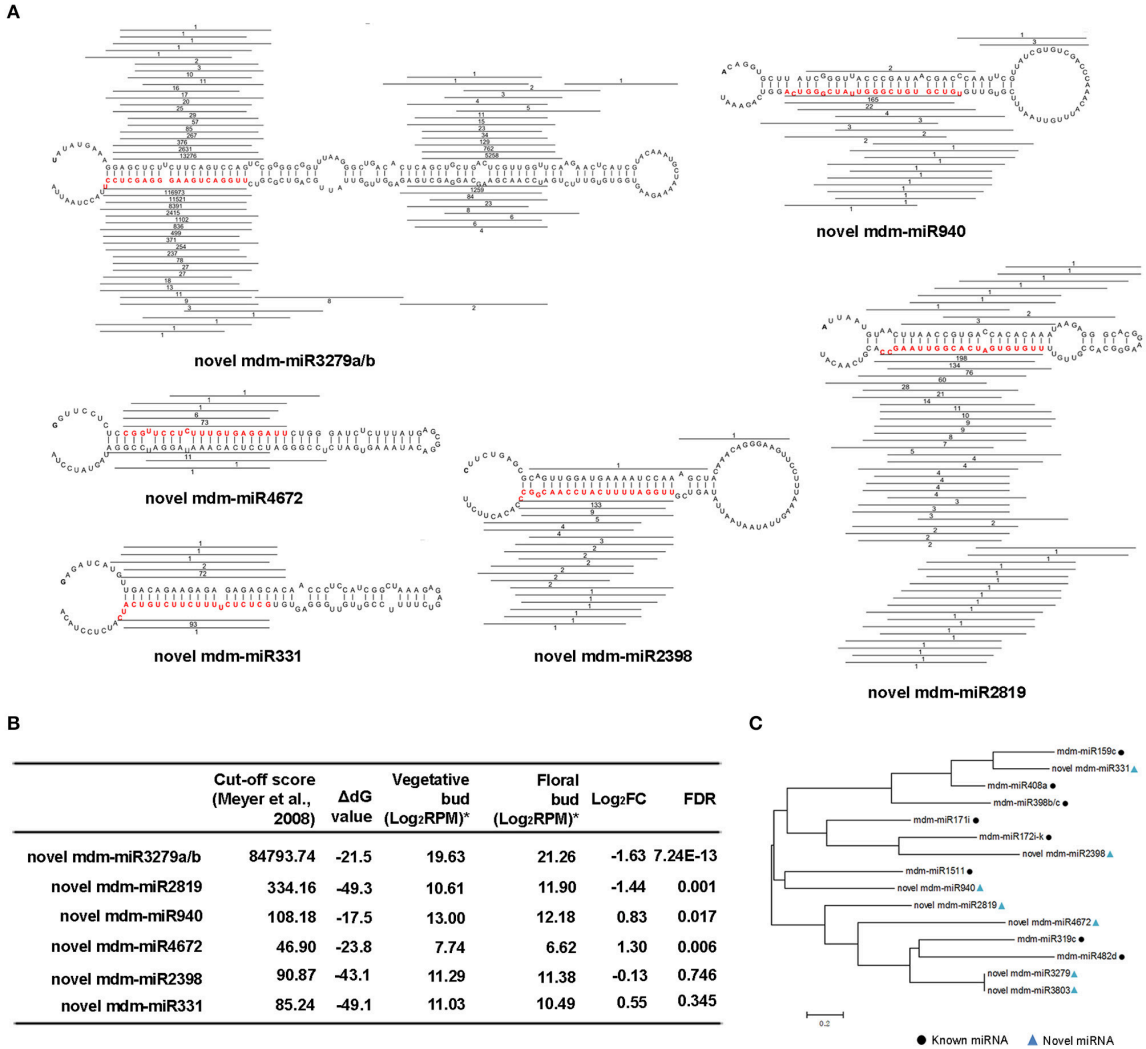
**Novel miRNAs that are correlated with floral transition from *Malus domestica*. (A)** Novel miRNAs and their secondary structures of pri-miRNAs. The sequence of miRNA was labeled in red. All sRNAs were matched onto pri-miRNAs and their read numbers were shown for each unique read. **(B)** Expression pattern of novel miRNAs. ^*^All the values were given as Log_2_CPM (Counts per million). **(C)** Phylogenic tree of novel miRNAs and some known miRNAs. The phylogenetic tree was constructed using the Neighbor Joining Method with the Mega 7.0 software. Black square indicated known miRNA and blue triangle indicated novel miRNA.

Next, we pursued more stringent criteria according to Meyers et al. (2008) as described in Methods. Only candidates having at least two characteristics of the above could be annotated as miRNAs. Under these filters, we narrow down the candidate lists and finally recovered 6 novel miRNA candidates with high confidence. According to mfold, we included 20–25 nt upstream or downstream of miRNA/miRNA^*^ sequence and predicted pri-miRNA secondary structures. We also mapped all sRNAs that are derived from the pri-miRNAs and found that the distribution patterns of the novel miRNA/miRNA^*^s were characteristic of the previously established ones in model plants (Figure 3A). Thus, these newly identified sRNAs are most likely *bona fide* miRNAs in the apple tree.

Among these 6 novel miRNAs, novel mdm-miR3279, and mdm-miR3803 were proved to be the same one as their sequences are identical. As such, we re-annotated them mdm-miR3279a and miR3279b. Only novel mdm-miR3279a/b, miR2819, miR940, and miR4672 were significantly different expressed miRNAs according to FDR < 0.05 (Figure 3B, Table S6). Phylogeny tree analysis showed the floral bud-enriched novel mdm-miR3279a/b/2819 are close to miR319c and miR482d, which were also expressed higher in floral bud (Figures 2A, 3C). On the other hand, the vegetative bud-enriched novel mdm-miR940/4672 showed high similarity with floral bud-enriched miR1511 and miR319c.

We then performed target prediction by PsRobot (Wu et al., 2012). As shown in Table 3, miR3279a/b target WD40 and MYB domain transcription factors that are correlated with anthocyanin biosynthesis and metabolic process; miR2819 may also target a transcription factor that is involved in metabolic process; miR2398 and miR940 repress the transcripts encoding factors that are engaged in protein-protein interaction; whereas miR4672 and miR331 repress genes that encode zinc binding protein.

**Table 3:** **GO analysis of potential targets of novel miRNAs during floral transition from *Malus domestica***.

**Name**	**Target protein**	**Protein ID**	**GO terms**	**GO ID**	**Pairing with target sequence (Upper line indicates miRNA, lower line indicates target)**
novel mdm-miR2398	Protein binding	MDP0000306873	Protein binding	GO:0005515	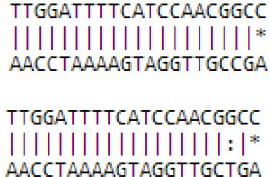
	Calcium-binding protein	MDP0000191848		GO:0008219 GO:0016021	
novel mdm-miR2819	Initiate or regulate RNA polymerase II	MDP0000288574	CaFBohydrate metabolic process	GO:0005975	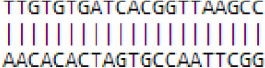
	Hydrolase	MDP0000447149	Polygalacturonase	GO:0004650	
	Transcription factor	MDP0000280817	Binding	GO:0005488	
novel mdm-miR3279/3803	Nucleus cellular componentwith WD40 domain	MDP0000251730	Protein binding	GO:0005515	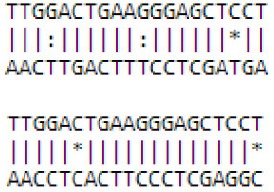
	Nucleus cellular componentwith MYB domain	MDP0000147309	Catalytic activity Metabolic processs DNA binding	GO:0003824 GO:0005515 GO:0008152 GO:0003677	
novel mdm-miR331	Zinc ion binding	MDP0000286463	ATP-dependent peptidase activity Proteolysis Zinc ion binding	GO:0004176 GO:0006508 GO:0008270	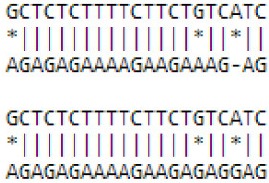
	Iron ion binding	MDP0000308925	Protein binding	GO:0005515	
novel mdm-miR4672	Iron ion binding	MDP0000161181	Iron ion binding Oxidoreductase activity	GO:0005506 GO:0016491	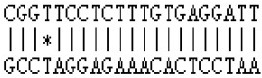
novel mdm-miR940	Protein binding	MDP0000281236	Protein domain specific binding	GO:0019904	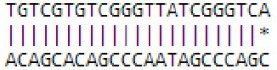

### 24-nt siRNAs in floral transition of apple trees

Increasing evidence has shown that chromatin methylation is closely related to flowering regulation in plants. siRNAs of 24 nt length are signatures of the RdDM pathway and play critical role in epigenetic silencing in plants (Matzke et al., 2015).

**Figure 4:**
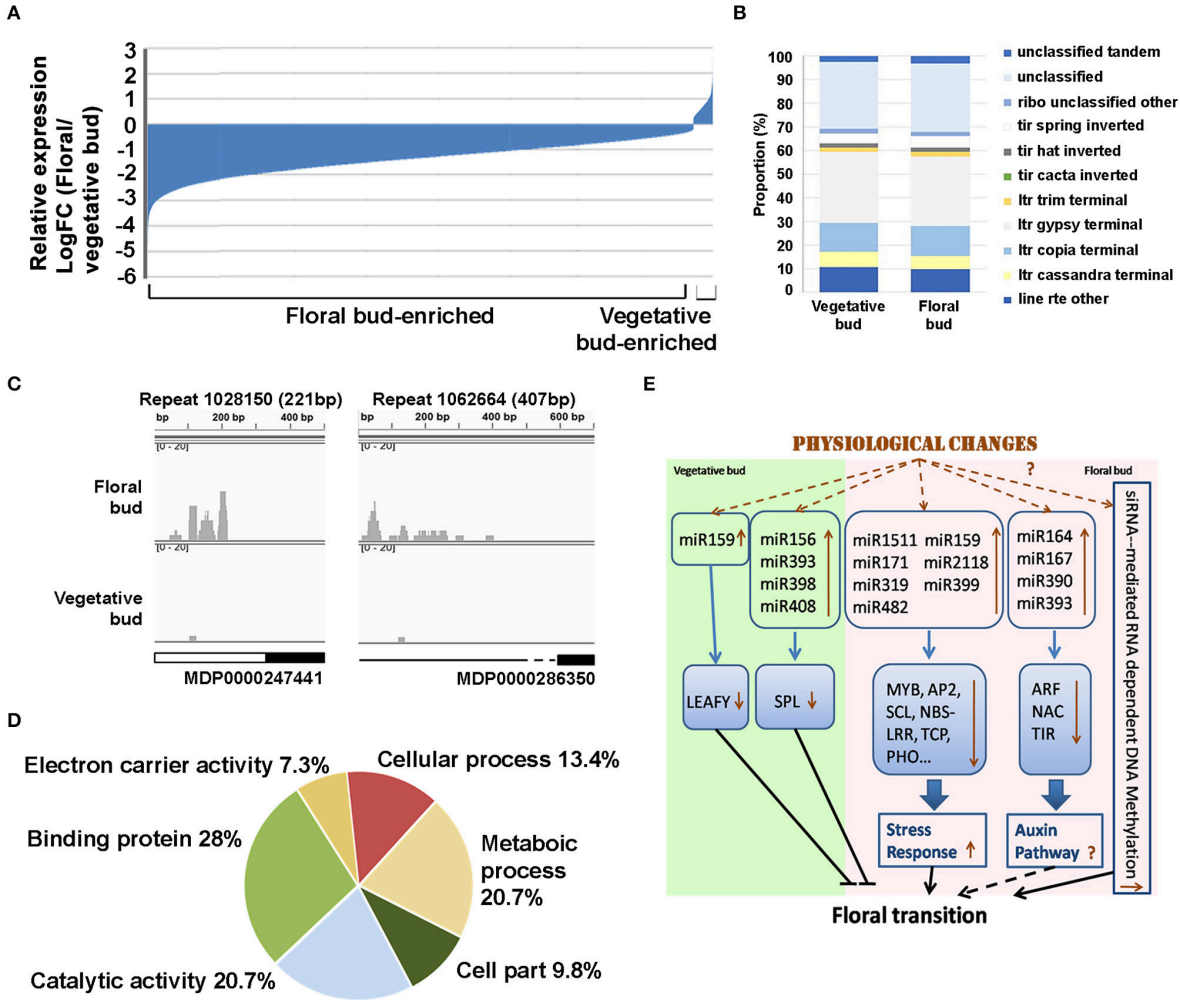
**Differentially expressed 24 nt-siRNA during floral transition of *Malus domestica*. (A)** Expression pattern of all differentially expressed siRNAs (DE-siRNAs) between vegetative and floral buds. Expression level was given as Log_2_Fold Change (FC, floral bud/vegetative bud). **(B)** Classification of all DE-siRNA genomic distribution. **(C)** IGV analysis of some top 50 DE-siRNAs. White box indicated intron, black box indicated extron and black line indicated intergenic region. Black imaginary line indicated the briefly long distance of intergenic region. **(D)** GO analysis of targeted loci by top 40 DE-siRNAs. **(E)** Potential model of sRNA-mediated floral transition in *Malus domestica*. Arrows indicated up-regulation or down-regulation. Dot line and “?” indicated unconfirmed interaction or terms.

We investigated the 24-nt sRNA population in vegetative and floral bud. 261, 702 differentially expressed (FDR < 0.05) 24-nt siRNAs were found; and among them, 2,469 were predominant in vegetative bud and 259, 233 were predominant in floral bud, which count for 9.4 and 90.6%, respectively (Figure 4A). This result suggests that DNA methylation status might be altered during the phase change in apple.

Among the DE-24nt-siRNAs, more than 60% were distributed over transposable element (TE) regions (Figure 4B), suggesting that a substantial amount of TEs are de-regulated in the floral buds compared to the vegetative buds. Then the top 40 differentially expressed 24 nt siRNAs (DE-siRNAs) screened by FDR < 0.05 were selected for further analysis (Table 4, Table S8).

**Table 4:** **Potential targets and TE elements of top 40 differentially expressed siRNAs during the floral transition of *Malus domestica***.

**Repeat**	**Chromosome**	**Start**	**End**	**TE element**	**Target protein ID**	**Target**
**INTRAGENIC REGION (INTRON)**						
repeat_1085094	Unanchored	51760264	51760412	LTR trim terminal	MDP0000664744	Heat shock protein HSP20
repeat_1117604	Chr2	10581879	10582001	Unclassified	MDP0000314081	Rhodanese-like protein
repeat_1028150	Chr3	32067196	32067416	LTR copia termial	MDP0000247441	Pectinesterase; DNA binding
repeat_1265460	Chr8	4276443	4276475	Unclassified tandem	MDP0000490697	Phosphatase
repeat_1265461	Chr8	4291702	4291734	Unclassified tandem	MDP0000488182	Unknown
repeat_853832	Chr9	30625201	30625309	TIR spring inverted	MDP0000438624	Unknown
					MDP0000266324	WD40-like protein
repeat_853530	Chr14	27263462	27263596	TIR spring inverted	MDP0000241035	Zinc ion binding
					MDP0000312371	ATP binding; nucleic binding
**INTRAGENIC REGION (EXTRON)**						
repeat_1270144	Chr2	4978879	4979011	Unclassified tandem	MDP0000197534	DNA binding
repeat_1039882	Chr4	22346821	22346934	Unclassified	MDP0000692845	Peptidase
repeat_1289450	Chr7	14280602	14280669	Ribosomal unclassified	MDP0000268430	Nucleus protein
repeat_1265459	Chr8	4285173	4285205	Unclassified tandem	MDP0000265846	Zinc ion binding
repeat_1267023	Chr9	30630665	30630773	Unclassified tandem	MDP0000321586	Isoprenoid synthase
repeat_1039981	Chr11	908937	909060	Unclassified	MDP0000849784	Unknown
repeat_1117835	Chr15	20360355	20360459	Unclassified	MDP0000304869	Unknown
repeat_1267817	Chr17	311713	311849	Unclassified tandem	MDP0000083486	ATase
repeat_853499	Chr17	5016279	5016409	TIR spring inverted	MDP0000286141	Glucosyltransferase
**INTERGENIC REGION**						
repeat_1269060	Unanchored	6189646	6189801	Unclassified tandem	MDP0000135966	Unknown
					MDP0000734661	Leucine-rich repeat
repeat_1270145	Chr2	4978251	4978383	Unclassified tandem	MDP0000197534	DNA binding
repeat_1039411	Chr4	1228773	1228888	Unclassified	MDP0000124365	Unknown
repeat_1269362	Chr4	1278056	1278216	Unclassified tandem	MDP0000872787	Unknown
repeat_1265373	Chr5	10388169	10388199	Unclassified tandem	MDP0000319295	protein binding
repeat_1267275	Chr7	1274745	1274848	Unclassified tandem	MDP0000183959	Galactose-like
repeat_1260363	Chr8	365188	365314	Unclassified	MDP0000376261	Unknown
repeat_1040200	Chr9	12254661	12254789	Unclassified	MDP0000311464	Oxidoreducta
repeat_873607	Chr10	24553191	24553235	LTR trim terminal	MDP0000289531	Calcium ion binding
repeat_1119810	Chr12	1189324	1189408	Unclassified	MDP0000257983	Unknown
repeat_1062664	Chr14	16793739	16794145	LTR gypsy terminal	MDP0000286350	Endopeptidase
repeat_1040003	Chr15	11077070	11077182	Unclassified	MDP0000197911	Protein kinase
repeat_1039784	Unanchored	9053589	9053706	Unclassified	MDP0000228072	Unknown
repeat_1265811	Unanchored	92494467	92494514	Unclassified tandem	MDP00001634	Actin-binding FH2
repeat_1265458	Unanchored	102659034	102659066	Unclassified tandem	MDP0000156554	Iron ion binding
repeat_1266082	Chr3	8503155	8503210	Unclassified tandem	MDP0000165108	Ribosome
					MDP0000152862	Unknown
repeat_853972	Chr3	8672342	8672447	TIR spring inverted	MDP0000252384	Unknown
repeat_854466	Chr8	4291702	4291734	Unclassified tandem	MDP0000209821	Iron ion binding
repeat_1270178	Chr10	12548160	12548315	Unclassified tandem	MDP0000649828	Calcium ion binding
repeat_855585	Chr12	29732429	29732584	TIR spring inverted	MDP0000214500	Diacylglycerol acyltransferase
repeat_1265663	Chr15	25979552	25979599	Unclassified tandem	MDP0000228862	Actin-binding FH2
repeat_1039352	Chr15	28620140	28620275	Unclassified tandem	MDP0000088811	Protein binding
repeat_856374	Chr16	7712559	7712712	TIR spring inverted	MDP0000307719	Glutamate_decaFBoxylase
repeat_854465	Chr17	311713	311849	Unclassified tandem	MDP0000203334	Protein binding

IGV analysis showed that except five siRNAs unanchored, the distribution of other top 40 DE-siRNAs almost covers all the 17 chromosomes of apple genome (Table 4). Among them, 28 DE-siRNAs were located at the intergenic regions and 17 DE-siRNAs located at the intragenic regions. As examples, repeat 1028150 was distributed at intron of MDP0000247441, and repeat 1062664 was distributed at intergenic region of MDP0000286350 (Figure 4C). These results suggested that siRNAs might regulate the expression of the loci flanking the siRNA producing regions during the developmental transition. GO analysis of potential targeted loci revealed that the genes affected by the top 40 DE-siRNAs mainly encode ion binding proteins, enzymes related to metabolic, catalytic, and cell processes, electron carrier and cellular parts (Figure 4D, Table 4, Table S9). These results indicated that these 24 nt-siRNAs might be involved in apple floral transition by targeting TEs that are distributed around the genes related to cellular signal transduction, cell growth, and metabolic process.

## Discussion

### Correlation of miRNA-mediated floral transition to *SPL* gene regulation, stress response, and auxin and GA pathways

In this study, we identified numerous known miRNAs that show differential expression patterns in floral transition during flower induction in apple trees. The numbers of DE miRNA members were comparable with the ones reported with peach vegetative buds, pear floral buds, apple floral bud (“Fuji”), and apple leaves (Barakat et al., 2012; Wu et al., 2014; Xing et al., 2014, 2016). These results suggest the conserved distribution of miRNAs among species.

In DE-miRNAs analysis, only miR398, miR408, miR159, and miR156 expressed stronger in vegetative bud. Among them, miR398, miR408, and miR156 are all related with the repression of *SPL* genes, a key factor in juvenile growth and a positive regulator of flowering process (Wu et al., 2009; Zhou C. M. et al., 2013). miR156 has been widely reported to targeted *SPLs*. Moreover, in *Citrus* trees, fewer fruit load in an OFF year (light yield) increases floral bud numbers and the expression of *SPLs* in floral bud during flower induction the following year, demonstrating the positive role of SPLs in flowering (Shalom et al., 2012). miR398 represented the miRNA with the most significant changes among all the DE-miRNAs. As reported, SPL7 could activate miR398 expression by binding to its core elements in the promoter (Yamasaki et al., 2009). This result is also consistent with the finding of a previous study where the expression of miR398 was significantly decreased in the floral buds in response to shoot bending, a technique used to promote flowering in apple (Xing et al., 2016). miR408 functions in copper homeostasis regulation together with miR398 in Arabidopsis and *Populus* (Lu et al., 2005; Abdel-Ghany and Pilon, 2008), and it also involves in the HY5-SPL7 network that mediates the coordinated response to light and copper (Yamasaki et al., 2009; Zhang et al., 2014). Besides, miR159 is regulated by GA pathway during flower development (Reyes and Chua, 2007). High expression of miR159 could reduce LFY activity and results in delayed flowering (Achard et al., 2004). Together, the above evidence indicate that in apple trees, miRNAs might control the vegetative growth through the regulation of *SPLs* and GA pathway (Figure 4E).

GO analysis showed floral bud-enriched DE-miRNAs mainly related with stress response and auxin signaling. The link between stress response and floral transition in plants has been demonstrated by previous studies. In Arabidopsis, constitutive expression of *ABI5*, which could positively response to ABA signaling, causes delay in flowering by up-regulating *FLC* expression (Wang et al., 2013). While drought stress at the beginning of the flowering results in early arrest of flowering by regulating genes including *DREB, MYB, VEGETATIVEY*, and *CO1*, etc. (Su et al., 2013). Here, the floral bud-enriched miR482 and miR2118 are involved in plant fungal pathogen resistance (Zhai et al., 2011); miR171 play roles in ABA and GA pathways during flower development (Ma Z. et al., 2014). miR319 participates in abiotic stress response and regulate cell proliferation (Zhou M. et al., 2013). miR1511 and miR2118 could also respond to drought stress (Pantaleo et al., 2010; Wu et al., 2015).

miR164, miR167, miR390, and miR393 were found to involve in auxin signaling. In Arabidopsis, miR167-targeted *ARF6/8* are activators of auxin-responsive genes and could accelerate flowering timing (Nagpal et al., 2005); while the miR390-TAS3-repressed *ARF2/3/4* are known to be repressors of auxin-responsive genes and flowering (Fahlgren et al., 2006). The miR393-targeted *TIR1* is also known to repress flowering in Arabidopsis (Chen et al., 2011). Thus, since all these four miRNAs expressed at higher levels in the floral buds, it is unable to conclude the final auxin changes during apple floral transition. Since apple is a woody perennial plant, the above conflict might indicate a more complex network of auxin regulations during its floral transition.

Together, we propose that apple floral transition might cause physiological stress responses in floral bud, and as such, the stress responsive miRNAs increase. The miRNAs involved in auxin pathways might be further induced by the stress responsive miRNAs or directly induced by physiological changes, and consequently stimulating downstream cascade of physiological changes through the floral transition (Figure 4E).

### Correlation of miRNA-mediated floral transition to phase transition

In perennial plants, there is one-time of phase transition (from juvenile to reproductive) and many times of floral transition (from vegetative to reproductive) in their life cycle. The phase transition also represents the first time of floral transition. In many previous studies in Arabidopsis and other plants, it has shown that the function of miR156 and miR172 in phase transition also include the repression/promotion of flowering process, respectively. In our results, miR156 was expressed at a higher level in the vegetative buds, suggesting that miR156 could also control vegetative growth. However, the difference of miR156 expression between the vegetative and the floral buds was not as significant as the ones during phase transition in apple and other woody plants (Wang J. W. et al., 2011; Xing et al., 2014). Thus, miR156 might differentially regulate juvenile phase and vegetative phase by its abundance: higher abundance might be important for juvenile phase, while lower abundance might be important for vegetative growth.

Then, we compared our data with previous sRNA sequencing data of apple phase transition. In addition to miR156 and miR172, numerous DE-miRNAs were also identified to involve in floral transition in apple trees. Among them, miR164, miR171, miR482, and miR5225 and several reported novel miRNAs enriched in floral bud and also expressed stronger in adult materials in the previous studies (Xia et al., 2012; Xing et al., 2014), indicating their conserved roles in apple flowering process.

Interestingly, the majority of DE-miRNAs identified in our study (miR162, miR167, miR390, miR393, miR396, miR398, miR408, miR535, miR1511, miR2118, miR3627, and miR7124) showed opposite expression patterns compared to the ones in the previous study on phase transition in apple trees (Xing et al., 2014). Moreover, many previously reported novel miRNAs also displayed opposite patterns in our study (Figure S1). Together, these results suggested that although there are some common mechanisms shared by phase transition and floral transition, many miRNAs might function in these two different processes by changing their abundance or showing opposite expression patterns. If so, how the opposite expression patterns of the miRNAs contribute to the regulation of phase transition or floral transition would be exciting topics in the apple developmental studies.

### Correlation between floral transition and siRNA guided DNA methylation

Floral transition in perennial tree is an integrate response of many factors including both environmental stimuli and plant phsiological changes. Accumulating evidence indicates that epigenetic mechanisms, including DNA methylation, play essential roles in the whole process of flower development, including bud stage. DNA methylation could involve in bud dormancy in azalea and floral bud differentiation in *Castanea sativa* (Santamaria et al., 2009; Meijon et al., 2010). siRNAs (24 nt) involve in epigenetic silencing by guiding DNA methylation. We have identified numerous DE-24 nt-siRNAs during the developmental transition from vegetative to floral bud. More than 90% of the DE 24 nt-siRNAs showed stronger expression in a whole genome pattern in the floral buds, suggesting an increased level of DNA methylation during floral transition (Figure 4A, Table 4). Our results were reminiscent of the previous observations in other plants. In Arabidopsis, increased DNA methylation levels have been observed during the transition from floral meristem to early flower stage (Yang H. et al., 2015). In radiate pine, the basal portion of the growing needles (<1 cm) of vegetative trees displayed 42% less of DNA methylation level than the same portions of trees that finish floral transition (Fraga et al., 2002). In *Castanea sativa*, vegetative tree shoots have the lowest level of DNA methylation (13.7%), while mature-tree shoots have a DNA methylation level of 15.0% (Hasbun et al., 2005). Such results were also demonstrated in the vegetative shoots and flower shoots of the chestnut tree (Hasbun et al., 2007). Thus, it appears that the floral transition is positively correlated with DNA methylation. Together, all these results suggested the siRNA might function in floral transition by increasing DNA methylation level. The detailed regulation and coordination of siRNA-mediated RdDM during floral transition need to be further investigated in the near future.

In conclusion, in this study we found that the classic model of miR156-miR172 in flowering appears to apply to floral transition of apple trees. Moreover, many other miRNAs, annotated or newly discovered, also appear to play critical roles during this process and showed different regulation with the ones in apple phase transition in previous study. These miRNAs may function in floral transition mainly through *SPL* genes, the response to stress stimuli and GA and auxin signaling. Last but not least, 24 nt siRNA mediated RdDM appears to be engaged in apple floral transition. Taken together, this study provides useful information and resource for future study on the roles of small non-coding RNAs in the floral transition in apple trees or other perennial plants.

## Author contributions

XG, LC, XZ, and TL designed research; XG and ZZ performed research; LC provided samples; ZM, ZZ, and XG performed bioinformatic analysis; XG, LC, and XZ wrote the paper.

### Conflict of interest statement

The authors declare that the research was conducted in the absence of any commercial or financial relationships that could be construed as a potential conflict of interest.
