# Supplementary figures and images for: Small RNA-Sequencing Links Physiological Changes and RdDM Process to Vegetative-to-Floral Transition in Apple
Source: Front Plant Sci. 2017 May 29;8:873. doi: 10.3389/fpls.2017.00873 (PMC5447065; doi:10.3389/fpls.2017.00873)

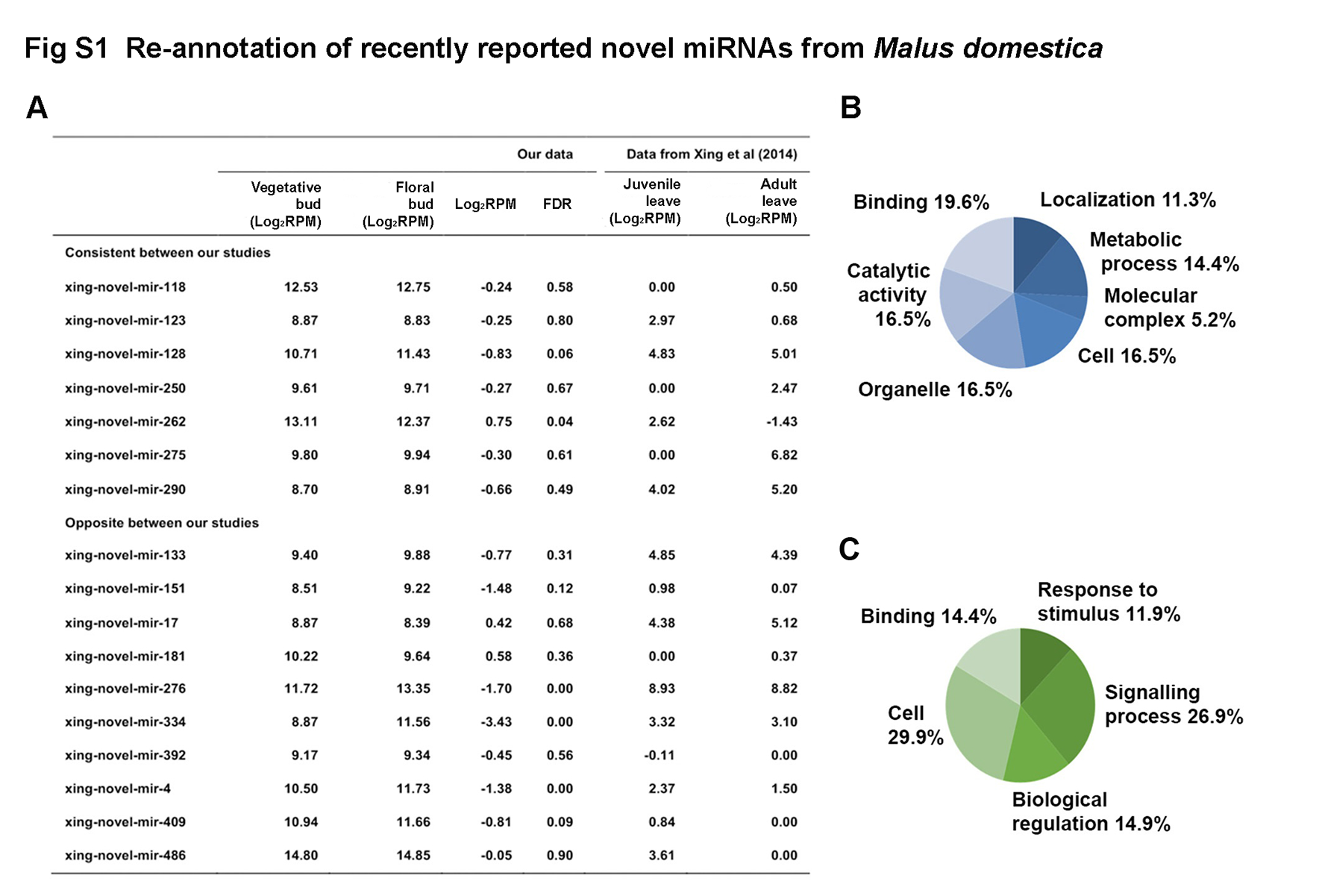

Supplement: Supplementary file 10 [file Image1.TIFF]
