# Supplementary material for: Small RNA-Sequencing Links Physiological Changes and RdDM Process to Vegetative-to-Floral Transition in Apple
Source: Front Plant Sci. 2017 May 29;8:873. doi: 10.3389/fpls.2017.00873 (PMC5447065; doi:10.3389/fpls.2017.00873)
Supplement: Supplementary file 11 [file Image2.pdf]

Figure S2. Secondary structures of 1st round screening of differentially expressed novel miRNAs from *Malus domestica* buds

noveliR_3627dG=-21.5

```
                  10        20             30
----------  tg  g      tga    --   a---|    g      g
          ag  gt tttttc   tcca  acg    agcag agccga a
          TC  CG GGGAAG   AGGT  tgc    tcgtt ttggtt g
taatatccat  CT  A      TC-    Tc   actg^    a      g
.        80        70         60        50        40
```

noveliR_3803dG=-21.5

```
                  10        20             30
----------  tg  g      tga    --   a---|    g      g
          ag  gt tttttc   tcca  acg    agcag agccga a
          TC  CG GGGAAG   AGGT  tgc    tcgtt ttggtt g
taatatccat  CT  A      TC-    Tc   actg^    a      g
.        80        70         60        50        40
```

noveliR_384dG=-101.4

```
            10        20        30        40        50
c----     g             CG      C                        c-|  c
     gggac gaaCGCCTCGTCC  TTCCGT CCGTcccgtcccacgtaccaaacg  acc t
     ccctg tttgcggagcagg  gaggca ggcagggtagggtgcatggtttgc  tgg a
ttgta     a             at      a                        ta^  c
 120       110       100        90        80        70        60
```

noveliR_2819dG=-49.3

```
        10         20        30        40        50        60        70
ccccttc   T-|     A              c            tatacctattacttttcg      tat
       tgt  TGTGTG TCACGGTTAAGCCa gtcaacatttta                  ttttat   \
       aca  acacac agtgccaattcggt cagttgtaaaat                  aaaata   t
gt-----   ct^     c              a            ttaattaa----------      tct
 .            120       110       100        90                  80
```

noveliR_493dG=-49.3

```
        10         20        30        40        50        60        70
ccccttc   T-|     A              c            tatacctattacttttcg      tat
       tgt  TGTGTG TCACGGTTAAGCCa gtcaacatttta                  ttttat   \
       aca  acacac agtgccaattcggt cagttgtaaaat                  aaaata   t
gt-----   ct^     c              a            ttaattaa----------      tct
 .            120       110       100        90                  80
```

noveliR_1008dG=-21.4

```
        10        20        30         40
attagc|  g  TCCCA  GG        aaat-   gaccctaa
      act GG     CC  GCGTGCCA     gtt        \
      tga cc     gg  cgcgcggt     cga        a
a-----^  g  -----  a-        gcatc   accatcta
                  70         60        50
```

noveliR_2036dG=-21.4

```
        10        20        30         40
attagc|  g  TCCCA  GG        aaat-   gaccctaa
      act GG     CC  GCGTGCCA     gtt        \
      tga cc     gg  cgcgcggt     cga        a
a-----^  g  -----  a-        gcatc   accatcta
                  70          60         50
```

noveliR_2195dG=-21.4

```
        10        20        30         40
attagc|  g  TCCCA  GG        aaat-   gaccctaa
      act GG     CC  GCGTGCCA     gtt        \
      tga cc     gg  cgcgcggt     cga        a
a-----^  g  -----  a-        gcatc   accatcta
                  70          60         50
```

noveliR_2361dG=-13.1

```
                    10        20            30
------------   a    ta   ct    ttc---     -| a
            gca gttt  tgg  tttc      tttga ct g
            CGT CGAG  ACC  Ggag      aaact ga c
ttgtatcaaaAC   G    CC   CT    taaaca     a^ g
       70        60        50        40
```

noveliR_4826dG=-14.1

```
        10        20        30            40
atcttc      G    A  T   GA      tt----|    ag
      cggaTC AACT CG AGT  CGTATT      atata  \
      gtttag ttgg gt tta  gtatag      tatat  t
ta----      g    a  c   a-      ctatct^    at
     80        70         60        50
```

noveliR_3990dG=-30.7

```
        10        20        30        40        50        60        70
ttaatcacatCCGTT|                 c  ttt    cag   ctatttatattc      aaa
               CATCGTACATCGTGCGGT ag   tcgt   gta            aatttt   t
               gtagcatgtagcatgcta tc   agta   cat            ttaaaa   a
---------------^                 a  ttt    a--   ------------      aaa
                110       100        90                      80
```

noveliR_2855dG=-21.7

```
        10        20        30        40
ggactggactA|    AAGG      C   A         tac
           GCTTC    ACTAAG TGG CTggctta   t
           tgaag    tgattc atc gatcgaat   a
tg---------^    ----      a   a         cag
           70            60        50
```

noveliR_786dG=-28.2

```
              10        20        30        40
t------|tg aa   t  t       aa      cat  tg    g  g
       g  a  tga gc caactag  gtagca   gt  cgag ct c
       t  T  ACT CG GTTGGTT  CGTTGT   cg  gttc gg t
gttgtgg^gt CG   -  -       C-      att  gt    -  c
 90       80          70         60         50
```

noveliR_2661dG=-28.2

```
              10        20        30        40
t------|tg aa   t  t        aa       cat  tg    g  g
       g  a  tga gc caactag  gtagca   gt  cgag ct c
       t  T  ACT CG GTTGGTT  CGTTGT   cg  gttc gg t
gttgtgg^gt CG   -  -        C-       att  gt    -  c
 90        80          70         60        50
```

noveliR_4520dG=-28.2

```
              10        20        30        40
t------|tg aa   t  t        aa       cat  tg    g  g
       g  a  tga gc caactag  gtagca   gt  cgag ct c
       t  T  ACT CG GTTGGTT  CGTTGT   cg  gttc gg t
gttgtgg^gt CG   -  -        C-       att  gt    -  c
 90        80          70         60        50
```

noveliR_940dG=-17.5

```
                 10          20        30
tt----------          --|  c   a   ttgttaa
            actcgataac  gac tga cac       t
            TGGGCTATTG  CTG GCT GTg       t
taaggactgtAC          GG^  T   -   ctgtrct
  70        60        50         40
```

noveliR_4937dG=-30.7

```
       10        20        30        40        50        60        70
ttaatcacatCCGTT|                  c  ttt    cag   ctatttatattc      aaa
               CATCGTACATCGTGCGGT ag   tcgt   gta             aatttt   t
               gtagcatgtagcatgcta tc   agta   cat             ttaaaa   a
---------------^                  a  ttt    a--   ------------      aaa
                110       100        90                       80
```

noveliR_4037dG=-30.7

```
       10        20        30        40        50        60        70
ttaatcacatCCGTT|                  c  ttt    cag   ctatttatattc      aaa
               CATCGTACATCGTGCGGT ag   tcgt   gta             aatttt   t
               gtagcatgtagcatgcta tc   agta   cat             ttaaaa   a
---------------^                  a  ttt    a--   ------------      aaa
                110       100        90                       80
```

noveliR_4703dG=-26.4

```
         10            20        30        40
attc   -   -   --|  TG        G     Cagtcgaat      ac
    ttg agg CCC  TGA  AGGTTTT CGGAG         ctttgt  \
    aac ttc ggg  gct  tccaaaa gcctc         gagacg  a
ga--   g   a   tc^  ca        -     cat------      cc
   90        80        70         60              50
```

noveliR_378dG=-26.4

```
         10            20        30        40
attc   -   -   --|  TG        G     Cagtcgaat      ac
    ttg agg CCC  TGA  AGGTTTT CGGAG         ctttgt  \
    aac ttc ggg  gct  tccaaaa gcctc         gagacg  a
ga--   g   a   tc^  ca        -     cat------      cc
   90        80        70         60              50
```

noveliR_4659dG=-26.4

```
        10            20         30         40
attc    -   -    --|  TG        G      Cagtcgaat      ac
    ttg agg CCC  TGA  AGGTTTT CGGAG         ctttgt  \
    aac ttc ggg  gct  tccaaaa gcctc         gagacg  a
ga--    g   a    tc^  ca        -      cat------      cc
   90         80         70          60               50
```

noveliR_2398dG=-43.1

```
              10         20         30         40         50
-------|   a     t                a      ttattataacttta    g
       gtgg ccgtt gatgaaaattcaacg ctataa              aagg a
       cacC GGCAA CTACTTTTAGGTTgc gatgtt              ttcc c
ttactca^   C     C                c      tg------------    c
 .         90         80         70         60
```

noveliR_1799dG=-17.1

```
           10         20         30         40
--       ct--|  a    tc   c  a    tttttct   t
  cgtggt    gca gtct  tgg at tctg       atc t
  gtatca    CGT CGGG  ACC TG agat       tag c
tt       aaAC^  G    TC   C  A    -------   t
         70         60         50
```

noveliR_331dG=-49.1

```
              10          20          30         40
c-------   t          -             a-|  rc  t   ct    ag
        atg tgacagaag agagagagcaca  ccc  ca cgg  aaag  \
        taC ACTGTCTTC TTTCTCTCGtgt  ggg  gt gcc  tttc  a
acatcctc   T          T             ga^  tt  t   t-    tg
 .         90         80         70         60          50
```

noveliR_4164dG=-37.4

```
        10            20         30         40         50
gagaaa   a   TG--  A-|     T          g  tcg   a  ggtg    ttcg
      gcc TCC    CT  GGGGTC GGCTCTCga at   gat gc    tctc    a
      cgg agg    ga  tctcgg ctgagggtt ta   cta tg    agag    t
gg----   g   ctta  gc^     t          g  ---   a  aa--    tttt
    110        100         90         80            70           60
```

noveliR_3146dG=-93.2

```
          10         20         30         40         50          60
--  c                               c          agc           -|  c
  tc agcttagtccttgaagctagtccagtc gagatagtcc   gcaacaaacgc ccc t
  ag tcgaaTCAGGGACTTCGATCAGGTCAG ctttgtcagg   cgttgtttgcg ggg t
tc  a                               A          cca           t^  a
 120        110        100         90         80         70
```

noveliR_2736dG=-16.4

```
          10         20         30
taa--|  aat         aaa   a    a    aa
     ttg   aaatggata   act ggga cttt  \
     agc   TTTACTTGT   TGG CCCT GAAa  c
caaaa^  aaT         CA-   -    C    ag
  70         60          50          40
```

noveliR_5130dG=-40.9

```
        10        20        30        40
ctca|         a           g            ttatattt
    tatttctgtg tcacggttaag tacgttaatatt        t
    ataaagataT AGTGCCAATTC GTGCAGTTGtaa        c
g---^         C           G            tttcgtta
     90        80        70        60        50
```

noveliR_4051dG=-13.4

```
        10            20        30        40        50
gaatcac     ---    tt-|  t    a     gtttaaa  tt  ccagtt
       ttgat   ttcg   gag attg agaac       gg  ac      t
       aactg   AGGC   CTT TGAC TTTTG       cc  tg      t
tttc---     aCC    TCC^  T    C     aacgg--  tt  agagac
 100           90        80        70          60
```

noveliR_4399dG=-49.5

```
        10        20        30           40        50        60
aaa                        a  a    aaa---|  tt   aattt   t      a
   tcacgtccgttcatcgtacatcgt cg ttag      tca  gta     ttt tattta a
   agtgtaggcaAGTAGCATGTAGCA GC AGTC      agt  cat     aaa ataagt a
---                        C  C    Aaaagc^  c-   gat--   t      t
    120       110       100        90         80          70
```

noveliR_4405dG=-29

```
             10         20           30
---     --    at  -          a    --|     aa
   tatgt  gagg  ga gagcttttgg ttgt  gagggt  g
   gtaca  TTCC  CT CTCGGGAATC GAta  ttcccg  g
taa     tt    C-  C          A    tt^     ga
80        70         60        50        40
```

noveliR_4255dG=-29

```
             10         20           30
---     --    at  -          a    --|     aa
   tatgt  gagg  ga gagcttttgg ttgt  gagggt  g
   gtaca  TTCC  CT CTCGGGAATC GAta  ttcccg  g
taa     tt    C-  C          A    tt^     ga
80        70         60        50        40
```

noveliR_1122dG=-49.4

```
        10        20        30         40        50        60
tgccc ty          ATC         C  -|                  tt     aaagataa
     t  tgTTTGTGTG   ACGGTTAAG ca gtcaacattttatattatt  tttat        t
     a  acaaacacat   tgccaattc gt cagttgtaaaatataataa  aagta        a
----- gt          caa         a  g^                  t-     aaaacaga
    130       120       110       100        90         80        70
```

noveliR_1077dG=-37

```
        10        20        30         40
gag|  atg    cg     g   tta   -     g  g  at--  tc
   gct   ctcg  gcgag cgt   ctc ggctg ag cg    gc  \
   cgg   gagc  CGCTC GTA   GAG TCGGC Tc gc    cg  a
---^  cg-    aT     -   TCG   A     -  g  ggag  tt
    90         80         70         60        50
```

noveliR_2426dG=-28

```
        10          20        30        40        50
acagta  ttG        --|        CCCTa          a   atg
      cc   GGCTTTTC  GTTAAAACT     gtttttaaagt cat   \
      gg   ccgaaaag  taattttgg     taagagtttta gta   a
gta---  ttg        at^        tgttc          g   gca
100           90        80        70        60
```

noveliR_1424dG=-19.7

```
        10          20         30        40
catc    ag  a-    - a     t  -|    ata   atattctca
    gcct  gg  agtg g tcctg aa gcctt   tgt         t
    tgga  tt  TCAC C AGGGT TT CGGAG   Aca         c
----    ct  gg    T A     T  C^    C--   aatccatrt
      90        80        70          60        50
```

noveliR_1225dG=-28

```
        10          20        30        40        50
acagta  ttG        --|        CCCTa          a   atg
      cc   GGCTTTTC  GTTAAAACT     gtttttaaagt cat   \
      gg   ccgaaaag  taattttgg     taagagtttta gta   a
gta---  ttg        at^        tgttc          g   gca
100           90        80        70        60
```

noveliR_175dG=-35.1

```
         10        20        30
-|  tcc   g    TG T      G         a  t
 cgg   gat GGCC  G CAGGAG ATCCTCTCc tc a
 gct   tta ttgg  t gttctc taggagagg ag c
t^  ---   g    gt -      -         -  c
  70           60          50         40
```

noveliR_1983dG=-58.9

```
               10        20        30        40        50        60
-------|         C                         aaa     t  attt        aaaat
       cacatcttaa CGTTCATCGTATATCGTGCGGTtag   tcatt ga    tattattt     t
       gtgtaggatt gtaagtagtatatagcacgccagtt   agtag ct    atgataaa     a
cctagga^         a                         aaa     t  ----        wacaa
 130       120       110       100        90        80             70
```

noveliR_4672dG=-57.1

```
        10        20        30         40        50
  ttc  c     T    C                -|   y       ay
gg   ct tcCGG TCCT TTTGTGAGGATTctgg gatc ctttatg  c
cc   ga aggcc agga aaacactcctagggcc ctag gaaatac  g
  tat  t     t    t                t^   t       ag
 .        90        80        70        60
```

noveliR_198dG=-12.3

```
        10          20         30
a      act--        t   a-  g    tt--|   tt
 tgattg     ttttaatg aaa  at tggt    ttcg  a
 attaat     GAAATTGC TTT  TA GCCA    aagt  a
a      cccTT        T   CG  G    taac^   ga
  80        70        60        50        40
```

noveliR_3686dG=-16.1

```
            10        20        30        40         50
---   c  -  aa      aaa             aaactgccagt-|   cttc   act
   tgg tt ag  cttaat   ttaattaaagagt            ttga    tga   a
   atc aa TC  GAATTA   GGTTAATTtttca            agct    act   t
tta   t  t  AG      AAA             aytccrctcttt^   ttca   cac
 .       110       100        90        80        70        60
```

noveliR_2828dG=-17.4

```
        10        20        30          40
tatatt|  c   C  AA  G  CCT      a   a--  t  g
      ggg TTC GC  CT GG  CATGAA  aat   ct gg g
      ctc aag cg  ga tc  gtattt  tta   ga tc g
t-----^  -   -  ag  g  at-      a   acc  t  a
      80          70         60        50
```

noveliR_4495dG=-18.5

```
        10         20        30        40
att    att         -|   A  CT  ACT    tcat
   gcca   GGTTTTATG GTGG AC  CA   ttct    g
   cggt   ccgaagtgt tatc tg  gt   aaga    g
ac-    aat         g^   c  tt  ac-    ttat
   80        70        60         50
```

noveliR_3959dG=-40.1

```
                 10          20             30        40
ac--------   tt -          --     -----|         gcatccatcc
          ggg  c acccgttagc  gacct     gacccgttaa          \
          CCT  G TGGGCAATTG  CTGGg     ttgggcaatt          a
gaccgtaaaa   GG T          GG     ttgag^         ataaacataa
 100        90        80        70        60        50
```

noveliR_1316dG=-51.1

```
               10        20        30        40
------       -|          C     T       Cacg   aagc
      gtgcctt cccGTGCTCTC TGTTT GTGTGGT    gtt    c
      cacggaa gggcacgggag acaaa cacacca    caa    a
gaagta       g^          a     -       ata-   ctgc
  90        80        70         60         50
```

noveliR_4201dG=-22.1

```
        10         20        30        40
ttttagca      C    -|    G  CCAaaat   gacc
        ctGTGT CCAC TGGGC TG       gtt    \
        gacgcg ggtg atccg ac       caa    c
atgagcca      c    c^    a  cat----   aaat
         70        60        50
```

noveliR_5060dG=-46.9

```
        10        20        30         40            50
cat| tttgg          T    T     - ct    tc        ----     tg
   tg     TAGCCAAGGA GACT GCCTg c  ccaa  acatgaat    ggcat  t
   ac     atcggttcct ctga cggac g  ggtt  tgtactta    ctgta  c
c--^ tcta-          -    -     c tc    --        aaat     tg
.          100         90         80          70        60
```

noveliR_4700dG=-20.2

```
             10        20        30
a-----|  g g       agatc  a      ag -  agaga
      gag c agttcag     ga aaggca  c tc     t
      ctc g TCGGGTC     CT TTTCGT  g ag     c
caaata^  - -       GAAAA  C      Ct c  agaag
 80         70        60        50        40
```

noveliR_1504dG=-20.2

```
        10        20        30              40
ctttg        -    CCT   A  TC       ta-----   aa  -| ga
     tcaagAAT TGAA   AAG TC  TCACTta       ggt  ag gg  \
     agttttta attt   ttt ag  ggtgaat       cca  tc cc  a
gtgta        t    tt-   a  tt       tatgatg   ga  a^ at
 100         90        80        70        60        50
```

noveliR_3314dG=-38.2

```
           10        20         30        40          50
c---    cga      CGAC    -|    GG       aggg--    g   t  aac
    tgtg   gtGTGC    AAGG ACGTC  GCCCcta      tggt gat gt   a
    acac   tacatg    ttcc tgtag  tgggggt      acca cta ca   t
actt    ccg      tata    g^    --       gaagca    a   -  ccc
 110       100        90          80        70         60
```

noveliR_1002dG=-25.3

```
             10        20         30        40
t---        --     G    C    -| C     ctgacga    ga
    ttcgcaaa  gCTGA TTTG GTGT GA GGGTG       ggct  \
    gagtgttt  tggct agat tata ct ctcac       tcga  g
gaac        gg     a    t    g^ t     ag-----    aa
 90        80        70        60             50
```

noveliR_866dG=-61.9

```
         10        20        30        40        50
-|                        C           ttattataa   t
 ggatccgggcCGTTGAATTTTGATC AACGGCTacag         ctt t
 cctaggcctggcaacttaaaactag ttgtcgatgtt         gaa a
c^                        a           tgtccctgg   a
 100        90        80        70        60
```

noveliR_734dG=-28.6

```
        10         20          30         40        50        60
aaaagg c       -  t        -- gt    -|       taatat      agactag
      g cttataa tg tagtaggt  g  gtcg gtttaata      agtgtc       \
      c gaatatt AC GTCATTTA  C  CAGT CAagttat      tcacag       a
g----- -       c  T        GG TG    A^       ------      cgttaac
       110       100        90        80              70
```

noveliR_5043dG=-32.2

```
        10         20        30                      40
cattc     ra-              a    a  y   --------------|   attt
     tctct   tttgtgtggtcacg ttga tc cgt              caac    \
     agaga   aaACACGCCAGTGT AATT AG GCa              gttg    t
-----     ata              C    C  T   attatagttacaat^   aata
      100        90        80        70        60        50
```

noveliR_4392dG=-25.2

```
                 10        20        30
-----    tt   ---|       GTCT   A     TT      ca
     tcac  tga   aGTGAGAG    TAG TTCGA  Ctcgt  \
     agtg  act   tcattctt    atc aagtt  gagcg  a
atcgg    tt   cga^       ac--   -     t-      ta
  80        70        60            50
```

noveliR_1224dG=-40.5

```
        10         20         30          40        50
gat  tg a  GG-      C-         G     c--|    accgc      tt
   gg  a tA   GATGGG  AACGGTTAT GCggg   aggta     ggttgt  a
   cc  t at   ttgccc  ttgccaata cgtcc   ttcgt     ccaata  c
at-  gt a  aga      at         -     ata^    attta      tc
 110        100        90         80        70         60
```

noveliR_3675dG=-51.2

```
          10        20        30        40        50
--|                     a     ttt    caggtac     a     aa
  gtccgttcatcgtatatcgtgc atcag   tcat       tattt tattt  t
  caggtaagTAGCATGTAGCACG TAGTC   agta       ataaa ataaa  t
ca^                     C     TTT    aaattt-     a     at
 110       100        90        80         70        60
```

noveliR_692dG=-40.5

```
        10         20         30          40        50
gat  tg a  GG-      C-         G     c--|    accgc      tt
   gg  a tA   GATGGG  AACGGTTAT GCggg   aggta     ggttgt  a
   cc  t at   ttgccc  ttgccaata cgtcc   ttcgt     ccaata  c
at-  gt a  aga      at         -     ata^    attta      tc
 110        100        90         80        70         60
```

noveliR_2578dG=-59.7

```
        10        20        30        40        50
gatc                            C           acaggg-|   ctt
    aagtgaTCCGGACCGTTGAAATTTGATC aacggttacaa       ggct   c
    tttactaggcctggtaactttaaattag ttgccgatgtt       ttga   t
----                            t           aataata^   aaa
     110       100        90        80        70        60
```

noveliR_3945dG=-60.8

```
          10        20        30        40
tttaa-|               C                 aaa  a
      tatgtGCTTATATGGT TTGGGCTACTCcccata   cc a
      atacacgaatatacca aacccgatgaggggtat   gg t
taaacg^               t                 aac  t
  90        80        70        60        50
```

noveliR_284dG=-28.3

```
            10        20          30        40
ggtaa---      t         -    act-|       t      g
        agtgaa agtacccgg attg    ttttagtg aaaaat \
        ttACTT TCGTGGGCC TGAC    gaaattgc tttttg t
ccttgaaa      T         A    AAGT^       -      g
  90        80        70        60          50
```

noveliR_1330dG=-22.2

```
           10        20          30        40
ggtaa---      t      a  -    act-|       t      g
        agtgaa agtacc gg attg    ttttagtg aaaaat \
        ttACTT TCGTGG CC TGAC    gaaattgc tttttg t
ccttgaaa      T      G  A    AAGT^       -      g
  90        80        70         60        50
```

noveliR_3897dG=-46.9

```
        10        20        30         40            50
cat| tttgg          T    T     - ct    tc        ----     tg
   tg     TAGCCAAGGA GACT GCCTg c  ccaa  acatgaat    ggcat  t
   ac     atcggttcct ctga cggac g  ggtt  tgtactta    ctgta  c
c--^ tcta-          -    -     c tc    --        aaat     tg
.          100         90         80          70        60
```

noveliR_4687dG=-23.1

```
           10        20        30        40
t- g    tt--|   ct     g        tg   a  aaataaag
  g gatt    tcca  aagcg ttattttc  gcg tg        a
  c ttaa    GGGT  TTTGC AGTGAAAG  Cgt ac        a
tg g    ttTC^   T-     -        GT   g  acgtgacg
  90        80          70        60        50
```

noveliR_790dG=-40.5

```
        10         20         30          40        50
gat  tg a  GG-      C-         G     c--|    accgc      tt
   gg  a tA   GATGGG  AACGGTTAT GCggg   aggta     ggttgt  a
   cc  t at   ttgccc  ttgccaata cgtcc   ttcgt     ccaata  c
at-  gt a  aga      at         -     ata^    attta      tc
 110       100        90         80        70        60
```

noveliR_2998dG=-101.1

```
        10        20        30        40        50        60
aca|     ta             ac             a                a    g
   tgggac  aacgcctcgtcct  tccgttccgtccc tcccacgtaccaaacg tacc t
   gccctg  tTGCGGAGCAGGG  AGGCAGGGCaggg agggtgcatggtttgc gtgg a
c--^     cc             CA             c                -    g
   120       110       100        90        80        70
```

noveliR_1586dG=-16.1

```
            10        20        30        40         50
---   c  -  aa      aaa             aaactgccagt-|   cttc   act
   tgg tt ag  cttaat   ttaattaaagagt            ttga    tga   a
   atc aa TC  GAATTA   GGTTAATTtttca            agct    act   t
tta   t  t  AG      AAA             aytccrctcttt^   ttca   cac
 .       110       100        90        80        70        60
```

noveliR_3292dG=-30.8

```
        10        20        30         40        50        60
at|  ta   aac    a   ac      a   -   taa     taataattwtat       a
  ttt  tcc   ggct caa  agagga tcc ctt   agtta            ccgttgy t
  agg  agg   TCGG GTT  TCTCCT AGG GGA   ttagt            ggcaact t
t-^  yg   aC-    -   CC      -   A   ---     tcttcacactc-       t
  120        110        100            90        80         70
```

noveliR_4245dG=-32.1

```
             10        20         30        40
cctc-----| a  at         aga-      tt t      t  r
         gg tc  tcaggcagt    tgatcg  c cagcca ct t
         cC AG  AGTCTGTTG    ACTGgt  g gtcggt ga t
tcaaccctt^ -  AC         GCAC      yg -      c  c
 90         80        70        60         50
```

noveliR_4924dG=-39.2

```
        10          20           30        40        50
agct       c       --      at--  -|   tccaat   ca       tat
    ggctgtt aggactc  tctggc    tc agcg      gtg  ggctcat   c
    tcggtag tTCTGAG  GGACTG    AG tcgt      cgc  ccgggta   a
g---       t       TT      CTCT  C^   ttttc-   aa       caa
  110       100        90        80         70        60
```

noveliR_4213dG=-33.7

```
          10        20        30        40        50
--| cc ga       CGA      CG   GG     aaga  g    at  t
  gg  g  agtGTGC   TGAGGA  TCG  CCCct    ga gtgg  tg \
  cc  c  ttatatg   attcct  agt  gggga    ct cacc  ac a
tc^ ta tc       taa      --   ga     cccg  a    ct  a
 100        90        80          70        60
```

noveliR_2179dG=-31.4

```
        10        20        30        40        50
atcacacaaa|   G     A   GAAG   acc       g     agaga
          ACAG AGGGC CGG    GGC   taaatgg agggc     \
          tgtc ttccg gtc    ccg   atttatt tcctg     a
----------^   g     -   aca-   gt-       -     aacct
          90         80          70        60
```

noveliR_4983dG=-57.2

```
        10        20        30        40        50        60
|   a    t            a          ca     tt     aggtac     a      a
ctgt aatt tgtccgttcatc tacatcgtgc  tcaga  ttatt      tgttt tgttta t
ggca ttag ACAGGCAAGTAG ATGTAGCACG  agtct  agtaa      ataaa ataaat t
^   c    c            C          cc     tt     gattt-     a      t
 130       120       110       100        90         80        70
```

noveliR_5045dG=-30.6

```
                      10        20        30
--------------                       tttt   -----|   tgt
              ggtctttcattcaaaaatatatc    agc     gaca   a
              TCGGAAAGTAAGTTTTTGtatag    tcg     ttgt   g
agcgtcgtgtGTAC                       t---   tacta^   tat
 90        80        70        60           50        40
```

noveliR_3641dG=-49

```
            10        20        30        40         50
----        aa                     a    tcayaag-|      ttc
    tattattg  actccaaaaatctcattctac ctcc        tgtattt   t
    ataataAC  TGAGGTTTTTAGAGTAAGAtg gagg        atataaa   t
ctta        CG                     y    tttgaaag^      cct
 110       100        90        80        70        60
```

noveliR_5039dG=-29.4

```
        10        20        30        40        50
cttatgtt|        GGC   GC     GC     ag t   g   t     ttat
        gcTTTGGTT   TCG  TTGTG  gttga  g gag gag ccctt    \
        tgagatcgg   agt  aatac  taact  c ctt ctc gggaa    a
--------^        gt-   a-     a-     -- -   g   -     taag
        100         90          80           70         60
```

noveliR_4444dG=-49

```
            10        20        30        40         50
----        aa                     a    tcayaag-|      ttc
    tattattg  actccaaaaatctcattctac ctcc        tgtattt   t
    ataataAC  TGAGGTTTTTAGAGTAAGAtg gagg        atataaa   t
ctta        CG                     y    tttgaaag^      cct
 110       100        90        80        70        60
```

noveliR_2265dG=-52.2

```
        10        20        30        40           50        60
cctca                          a  a    aaa---|  tt   aattt   t      a
     aatcacgtccgttcatcgtacatcgt cg ttag      tca  gta     ttt tattta a
     ttagtgTAGGCAAGTAGCATGTAGCA GC agtc      agt  cat     aaa ataagt a
gaaa-                          C  c    aaaagc^  c-   gat--   t      t
 130        120       110       100        90         80          70
```

noveliR_3679dG=-24

```
         10        20        30        40        50
-|    t  g               ata    aaaa     taaaattaaac
 gtgta ct tttattgtacattgt gtta      ttatt           a
 cacat ga agATAGCATGTAGCA CAGT      aatag           c
g^    -  -               CGC    CAAa     tccataayaaa
 100          90        80        70        60
```

noveliR_4959dG=-45.8

```
        10        20        30        40         50
gaag       a     c                      tc  aag-|      ttc
    tgttatt gtact taaaaatctcattctacacttc  ac    tgtattt   t
    acaataa cgTGA GTTTTTAGAGTAAGATGTGagg  tg    atataaa   t
----       c     A                      tt  aaag^      cct
     110       100        90        80        70        60
```

noveliR_2990dG=-36

```
                  10        20        30        40        50         60
----------|                a      a  cg  t   tttcgtcag   ctg-   a    caa
          aatcacattcgttcatc tacatt tg  gt agt         gta    ttt tatt   t
          TTAGTGTAGGCAAGTAG ATGTAa ac  ca tca         cat    gaa ataa   t
ctttagaagt^                C      a  at  -   tttaataa-   ttaa   a    att
 130       120       110       100         90         80        70
```

noveliR_1687dG=-49

```
            10        20        30        40         50
----        aa                     a    tcayaag-|     ttc
    tattattg  actccaaaaatctcattctac ctcc        tgtattt   t
    ataataAC  TGAGGTTTTTAGAGTAAGAtg gagg        atataaa   t
ctta        CG                     y    tttgaaag^     cct
 110       100       90        80        70        60
```

noveliR_677dG=-46.1

```
        10         20        30        40        50        60        70
tcgttt         ta-| a           c                    ctatttctttttg      tat
      ttctgtttt   tg tcacggttaag cgcgtcaatattttatatta             ttttat   \
      aggacaaaa   AC AGTGCCAATTC GTGCAGttataaaatataat             aaaata   t
------         cAC^ C           A                    aa-----------      ttt
       130       120       110       100        90                   80
```

noveliR_3790dG=-21.3

```
        10       20        30         40
t| tt    tac t    tt   c       g    -   aa
 tg  ccaa   g cact  tac gtgtggg ttgt gca  a
 ac  ggtt   C GTGA  GTG CGTATTT AGCa cgt  g
c^ t-    ttC -    T-   T       G    a   ca
 80          70         60        50
```

noveliR_3756dG=-33.6

```
        10        20        30         40        50        60
ccta   a         a     gt   ata    -|      cact   a       cttat
    ttt tgcggtcac gttaa  cac   aaca tttatat    att tttttgt     t
    aaa acgCCAGTG CAATT  GTG   TTGt aaatata    taa aaaaata     a
ata-   c         C     TG   CAG    a^      ac--   -       tatct
  120       110       100        90        80           70
```

noveliR_1837dG=-33.6

```
        10        20        30         40        50        60
ccta   a         a     gt   ata    -|      cact   a       cttat
    ttt tgcggtcac gttaa  cac   aaca tttatat    att tttttgt     t
    aaa acgCCAGTG CAATT  GTG   TTGt aaatata    taa aaaaata     a
ata-   c         C     TG   CAG    a^      ac--   -       tatct
  120       110       100        90        80           70
```

noveliR_4571dG=-30.1

```
             10        20           30        40        50
cactcc-----  a    ag   t  aa   ---|   t   aaaa          tat
           gg acgt  tgg tg  acc   atgt gag    atcatgaaca   \
           CC TGCG  GCC AC  TGG   tata ctc    tagtacttgt   g
cgtttgtaaaA  G    G-   -  CC   gaa^   c   aatc          tag
       100         90         80        70        60
```

noveliR_551dG=-49

```
            10        20        30        40         50
----        aa                     a    tcayaag-|     ttc
    tattattg  actccaaaaatctcattctac ctcc        tgtattt   t
    ataataAC  TGAGGTTTTTAGAGTAAGAtg gagg        atataaa   t
ctta        CG                     y    tttgaaag^     cct
 110       100       90        80        70        60
```

noveliR_170dG=-55.2

```
        10        20        30        40        50        60
|   c      c    a       a                   c   a  t   gtttg      a
tcct atatct atcc tttatcg acattgtgtgaccagaaat att tg att     tgttta t
agga tgtaga TAGG AAGTAGC TGTAGCACGctggtttttta taa ac taa     ataaat t
^   -      A    C       A                   a   a  t   aaa--      t
 130        120       110       100        90        80          70
```

noveliR_838dG=-43.1

```
               10        20        30        40        50
-------|   a     t               a      ttattataacttta    g
       gtgg ccgtt gatgaaaattcaacg ctataa              aagg a
       cacC GGCAA CTACTTTTAGGTTgc gatgtt              ttcc c
ttactca^   C     C               c      tg------------    c
 .        90        80        70        60
```

noveliR_5038dG=-29.4

```
        10        20        30        40        50
cttatgtt|        GGC   GC     GC     ag t   g   t     ttat
        gcTTTGGTT   TCG  TTGTG  gttga  g gag gag ccctt    \
        tgagatcgg   agt  aatac  taact  c ctt ctc gggaa    a
--------^        gt-   a-     a-     -- -   g   -     taag
        100         90          80            70         60
```

noveliR_220dG=-30.6

```
           10         20          30        40        50
c---  att        G-      -    CT-|      atatgtacy    carct
    ac   gcccAGGG  AGTGGA TCCT   AAGCCTt         tgct     \
    tg   tgggtttc  tcactt aggg   ttcggag         acga     c
agga  cct        gy      g    ttt^      c--------    mcctt
 100        90        80        70                 60
```

noveliR_3054dG=-24.4

```
        10        20        30
caggttccga| G   GC        C          gca
          CT ATT  TTCCTTT TCCTtgttct   \
          gg tag  aaggaaa gggaacgaga   g
g---------^ a   ga       c          acg
.                 60        50
```

noveliR_1748dG=-49

```
            10        20        30        40         50
----        aa                      a    tcayaag-|      ttc
    tattattg  actccaaaaatctcattctac ctcc        tgtattt   t
    ataataAC  TGAGGTTTTTAGAGTAAGAtg gagg        atataaa   t
ctta        CG                      y    tttgaaag^      cct
 110       100        90        80        70        60
```

noveliR_2233dG=-49

```
            10        20        30        40         50
----        aa                      a    tcayaag-|      ttc
    tattattg  actccaaaaatctcattctac ctcc        tgtattt   t
    ataataAC  TGAGGTTTTTAGAGTAAGAtg gagg        atataaa   t
ctta        CG                      y    tttgaaag^      cct
 110       100        90        80        70        60
```

noveliR_4470dG=-58.1

```
         10        20        30        40        50        60
gt-|                   a                  cttattac       tttat
   tttgtgtggtcacggttaagc acgtcaacattttatatt        tttttgt     t
   aaacacaCCAGTGTCAATTCG TGCAGTTGtaaaatataa        aaaaata     a
tac^                   G                  cwaa----       ttttt
 120       110       100        90        80            70
```

noveliR_607dG=-21.4

```
        10        20        30         40
attagc|  g  TCCCA  GG        aaat-   gaccctaa
      act GG     CC  GCGTGCCA     gtt        \
      tga cc     gg  cgcgcggt     cga         a
a-----^  g  -----  a-        gcatc   accatcta
                  70         60        50
```

noveliR_1136dG=-49

```
             10        20        30        40         50
----         aa                   a    tcayaag-|      ttc
    tattattg  actccaaaaatctcattctac ctcc        tgtattt   t
    ataataAC  TGAGGTTTTTAGAGTAAGAtg gagg        atataaa   t
ctta         CG                   y    tttgaaag^      cct
 110       100        90        80        70        60
```

noveliR_145dG=-52.2

```
        10        20        30        40           50        60
cctca                          a  a    aaa---|  tt   aattt   t      a
     aatcacgtccgttcatcgtacatcgt cg ttag      tca  gta     ttt tattta a
     ttagtgTAGGCAAGTAGCATGTAGCA GC agtc      agt  cat     aaa ataagt a
gaaa-                          C  c    aaaagc^  c-   gat--   t      t
 130        120       110       100        90        80           70
```

noveliR_2853dG=-49.7

```
        10        20        30        40        50        60
tcctg|    a         a                           ctattca       cttat
     tttgt tggttatg ttaagccacgttaacattttatatta       tttttgt     t
     aaaca aCCAGTAC AATTCGGTGCAGTTGtaaaatataat       aaaaata     a
cca--^    c         C                           -------       tctct
   120       110       100        90        80                70
```

noveliR_4373dG=-23.1

```
              10        20          30        40
t-----     ac-|    CCC    G    --     C aa    tgac    aaaa
      ttagc    ttAGT   ACT GGC  GTGC A  atgt    ctta    \
      aatcg    aatca   tga ccg  cacg t  taca    gaat    a
tgcatc     agt^    a--    g    ga     - gg    tcc-    catc
        90        80          70         60        50
```

noveliR_4832dG=-21

```
             10         20        30
ac------| a tg    g   c-       cgt   tta
        ac c  cgag ctg  tcggct   ggc   \
        tg G  GTTC GAC  GGTTGG   ccg   t
tggaagtt^ C GT    -   TC       Tt-   ttg
.        60         50        40
```

noveliR_248dG=-21.3

```
        10        20        30         40
t| tt    tac t    tt   c       g    -   aa
 tg  ccaa   g cact  tac gtgtggg ttgt gca  a
 ac  ggtt   C GTGA  GTG CGTATTT AGCa cgt  g
c^ t-    ttC -    T-   T       G    a   ca
 80          70         60        50
```

noveliR_249dG=-21.3

```
        10        20        30         40
t| tt    tac t    tt   c       g    -   aa
 tg  ccaa   g cact  tac gtgtggg ttgt gca  a
 ac  ggtt   C GTGA  GTG CGTATTT AGCa cgt  g
c^ t-    ttC -    T-   T       G    a   ca
 80          70         60        50
```

noveliR_2345dG=-21.3

```
        10        20        30         40
t| tt    tac t    tt   c       g    -   aa
 tg  ccaa   g cact  tac gtgtggg ttgt gca  a
 ac  ggtt   C GTGA  GTG CGTATTT AGCa cgt  g
c^ t-    ttC -    T-   T       G    a   ca
 80          70         60        50
```

noveliR_2346dG=-21.3

```
        10        20        30         40
t| tt    tac t    tt   c       g    -   aa
 tg  ccaa   g cact  tac gtgtggg ttgt gca  a
 ac  ggtt   C GTGA  GTG CGTATTT AGCa cgt  g
c^ t-    ttC -    T-   T       G    a   ca
 80          70         60        50
```

noveliR_1281dG=-37

```
        10        20        30         40
gag|  atg    cg     g   tta   -     g  g  at--  tc
   gct   ctcg  gcgag cgt   ctc ggctg ag cg    gc  \
   cgg   gagc  CGCTC GTA   GAG TCGGC Tc gc    cg  a
---^  cg-    aT     -   TCG   A     -  g  ggag  tt
    90         80         70         60        50
```

noveliR_2963dG=-46.4

```
        10        20          30        40            50
taacgag          G     AT--          t  tat     ----|     ca
       taaCGGGTCG GTCAT    TACCTGTTaa at   cgggt    tgattt  g
       attgcccagc cagta    gtgggcaatt ta   gccca    actggg  g
taga---          a     catt          t  ttt     tttt^     ct
 110          100        90        80        70        60
```

noveliR_3243dG=-38.7

```
                     10        20        30            40
-------------                         attwaawtttt----|    w
             cacggttaagctacgtcaacatttt               ttttt a
             GTGCCAATTCGGTGCAGTTGtaaaa               aaaaw w
acaaaacgcgTCA                         gaaaaatayaataat^    a
 100        90        80        70        60        50
```

noveliR_3142dG=-45.8

```
        10        20        30        40         50
gaag       a     c                     tc  aag-|      ttc
    tgttatt gtact taaaaatctcattctacacttc  ac    tgtattt   t
    acaataa cgTGA GTTTTTAGAGTAAGATGTGagg  tg    atataaa   t
----       c     A                     tt  aaag^      cct
      110       100        90        80        70        60
```

noveliR_4887dG=-37.1

```
            10        20        30        40        50
----|       ac          a   w           aa  tttc       gaa
    tgttattg  actttaaaaa ttt ttttacattcc  ac    tatattt   a
    acaataAT  TGAGGTTTTT GAG AAGAtgtgagg  tg    atataaa   a
cttc^       CA          A   T           ag  tta-       aag
 110       100        90        80        70         60
```

noveliR_1516dG=-20.2

```
        10        20        30         40        50
ccgcacaaatA|   GG  TGGA        a-   c   ga    agaca
           GGAG  CA    AGGGCACc  caa tag  gggc     \
           tttt  gt    tcttgtgg  gtt att  cctg     a
-----------^   aa  ttg-        ga   a   aa    aacct
          90        80         70        60
```

noveliR_9896dG=-36.7

```
                   10        20        30        40        50
ca-----------|   a                a   gtttttgtcaagt       g      a
             tcgt cattgtacatcgtgcg tca             agtgttt tgttta t
             GGCA GTAGCATGTAGCATGt agt             ttataaa ataaat t
ggcagttagaACA^   A                c   atttaaawaaawt       a      t
 120       110       100        90        80        70        60
```

noveliR_662dG=-57.2

```
        10        20        30        40        50        60
|   a    t            a          ca     tt     aggtac     a      a
ctgt aatt tgtccgttcatc tacatcgtgc  tcaga  ttatt      tgttt tgttta t
ggca ttag ACAGGCAAGTAG ATGTAGCACG  agtct  agtaa      ataaa ataaat t
^   c    c            C          cc     tt     gattt-     a      t
 130       120       110       100        90        80        70
```

noveliR_1163dG=-40.3

```
         10        20        30        40                             50
-           c  a      atc          a        --------------------|     t
 attttgtgtgg ca ggttaa   acgtcaacat ttatattg                    attttt t
 taaaacacgCC GT CCAATT   TGCAGTTGta aatataat                    tagaga a
a           A  G      CGA          a        gataacaaaaaacagaataa^     t
 120       110       100        90        80        70        60
```

noveliR_2507dG=-25.8

```
        10        20        30        40         50
gaag       a     c                     tc  aag-|      ttc
    tgttatt gtact taaaaatctcattctacacttc  ac    tgtattt   t
    acaataa cgTGA GTTTTTAGAGTAAGATGTGagg  tg    atataaa   t
----       c     A                     tt  aaag^      cct
      110       100        90        80        70        60
```

noveliR_317dG=-17.4

```
                10         20        30
t--------       aa   agtg-   aga     ca---|     ga
         cttcatc  cca     atc   tgtct     ggaact  t
         gAGGTGG  GGT     TGG   acgga     ttttga  g
tacggggtt       A-   GGAGG   aa-     ttaag^     ac
       80         70        60         50        40
```

noveliR_964dG=-45.8

```
        10        20        30        40         50
gaag       a     c                      tc  aag-|      ttc
    tgttatt gtact taaaaatctcattctacacttc  ac    tgtattt   t
    acaataa cgTGA GTTTTTAGAGTAAGATGTGagg  tg    atataaa   t
----       c     A                      tt  aaag^      cct
     110       100        90        80        70        60
```

noveliR_1409dG=-45.1

```
        10        20        30        40        50        60
|  c     a  a          c        c   caattt    cyagt   a   a      a
cct aaatc tg ccgttcatcg atatcgtg ygt      tcat     yta ttt tattta t
gga tttag AC GGCAAGTAGC TGTAGCAC cca      agta     tat gaa ataaat t
^  a     c  A          A        A   atrrtt    aaatt   -   a      t
 130       120       110       100        90        80         70
```

noveliR_2572dG=-36.7

```
                   10        20        30        40        50
ca-----------|   a                a   gtttttgtcaagt       g      a
             tcgt cattgtacatcgtgcg tca             agtgttt tgttta t
             GGCA GTAGCATGTAGCATGt agt             ttataaa ataaat t
ggcagttagaACA^   A                c   atttaaawaaawt       a      t
 120       110       100        90        80        70        60
```

noveliR_1399dG=-45.8

```
        10        20        30        40         50
gaag       a     c                      tc  aag-|      ttc
    tgttatt gtact taaaaatctcattctacacttc  ac    tgtattt   t
    acaataa cgTGA GTTTTTAGAGTAAGATGTGagg  tg    atataaa   t
----       c     A                      tt  aaag^      cct
     110       100        90        80        70        60
```

noveliR_24dG=-49

```
            10        20        30        40         50
----        aa                     a    tcayaag-|      ttc
    tattattg  actccaaaaatctcattctac ctcc        tgtattt   t
    ataataAC  TGAGGTTTTTAGAGTAAGAtg gagg        atataaa   t
ctta        CG                     y    tttgaaag^      cct
 110       100         90        80        70        60
```

noveliR_3193dG=-26.8

```
        10         20           30           40
atgtttggttA        -   --  C-    G   at---|       c
           GGAGGATC CTC  TC  TTGG CTC     gaggggat c
           ctttttag gag  ag  aact gag     tttcccta a
cgtatga----        a   tt  tt    g   gatat^       a
  90            80        70        60        50
```

noveliR_3071dG=-18

```
        10        20         30        40
tacactaagg    GG     -      CCTc          ag--| at
          GGAG  GGTGG GCTTAG    ataatttgtt    ca  a
          tctc  ttatt tgaatt    tgttgaataa    gt  a
ta--------    tt     a      att-          ctta^ gt
.                80        70         60        50
```

noveliR_39dG=-49

```
            10        20        30        40         50
----        aa                     a    tcayaag-|      ttc
    tattattg  actccaaaaatctcattctac ctcc        tgtattt   t
    ataataAC  TGAGGTTTTTAGAGTAAGAtg gagg        atataaa   t
ctta        CG                     y    tttgaaag^      cct
 110       100        90        80        70        60
```

noveliR_2221dG=-17.9

```
            10             20        30
tggaaac----       -    ----|    aa   t
           ggtgtcg ttgt    agtaa  ttg t
           CCGCGGC GACA    TCgtt  gac a
gtagttgtaaA       A    CCCT^    ac   g
        60        50        40
```

noveliR_4196dG=-49.1

```
        10         20        30        40        50        60        70
tcgttt         ta-| a           c                    ctatttctttttg      tat
      ttctgtttt   tg tcacggttaag cgcgtcaatattttatatta             ttttat   \
      aggacaaaa   AC AGTGCCAATTC GTGCAGttataaaatataat             aaaata   t
------         cAC^ C           A                    aa-----------      ttt
       130       120       110       100        90                    80
```

noveliR_4454dG=-16.1

```
        10        20         30        40
cctcagamttC| T  C   T       -   Ctat   aag     g
           GT CA TGT TTTCATG GCC    tgg   attca a
           ca gt ata aaggtat cgy    gcc   tgggt t
a----------^ -  -   t       t   c---   gtg     a
           80          70           60        50
```

noveliR_5013dG=-44.5

```
        10        20        30                    40         50
tccc        a                   -----------   g-       tt-|       cta
    gtgatcat accgtttattgtatatcgt           tca  ttagttt   gttaggta   \
    cactagta TGGCAAGTAGCATGTAGCA           agt  aattaaa   taatttat   t
c---        c                   CGCCatttttt   aa       ttt^       att
    120       110       100        90        80        70        60
```

noveliR_1028dG=-17.7

```
            10        20        30        40
--      ct--|  a    tc   c  a    tttttct   t
  cgtggt    gca gtct  tgg at tctg       atc t
  gtatca    CGT CGGG  ACC TG agat       tag c
tt      aaAC^  G    TC   C  A    -------   t
        70        60        50
```

noveliR_3369dG=-58.1

```
         10        20        30        40        50        60
gt-|                    a                  cttattac       tttat
   tttgtgtggtcacggttaagc acgtcaacattttatatt        tttttgt     t
   aaacacaCCAGTGTCAATTCG TGCAGTTGtaaaatataa        aaaaata     a
tac^                    G                  cwaa----       ttttt
 120       110       100        90        80            70
```

noveliR_3306dG=-19.4

```
           10        20        30                 40
a---  gr  tg     ta  c        tttt-     --------|     t
    gt  tt  accga  gt acctctat     taaaa        ttaaaa a
    cg  ag  TGGTT  CG TGGAGATA     atttt        agtttt a
tgaa  gt  CG     CA  C        cgtct     actattta^     t
.        90        80        70        60        50
```

noveliR_936dG=-52.2

```
         10        20        30        40           50        60
 cctca                          a  a    aaa---|  tt   aattt   t      a
      aatcacgtccgttcatcgtacatcgt cg ttag       tca  gta     ttt tattta a
      ttagtgTAGGCAAGTAGCATGTAGCA GC agtc       agt  cat     aaa ataagt a
 gaaa-                          C  c    aaaagc^  c-   gat--   t      t
  130        120       110       100        90         80          70
```

noveliR_1409dG=-63.9

```
           10        20        30        40        50         60
---|                       a      c  ttt     aggtact     t-      tttt
   gatcgtgaccgttcatcgtacatc tgcggt ag   tcgtt       attta  atttaa    \
   ctagcacTGGCAAGTAGCATGTAG ACGCCa tc   agtaa       taagt  taaatt    a
gca^                       C      a  ttt     agttt--     tt      ttaa
 130       120       110       100        90          80        70
```

noveliR_4144dG=-21.3

```
        10        20        30         40
t| tt    tac t    tt   c       g    -   aa
 tg  ccaa   g cact  tac gtgtggg ttgt gca  a
 ac  ggtt   C GTGA  GTG CGTATTT AGCa cgt  g
c^ t-    ttC -    T-   T       G    a   ca
 80          70         60        50
```

noveliR_2430dG=-39.4

```
        10          20        30        40        50        60
atta  a   -    -| ACC       CCAaaat      cc      c   aga    a  tg
    gc ctg GATC CC   GGGCGTG       gttagt  tagaaa tac   gcct cg  \
    cg ggc ttag gg   cctgcat       caatcg  atcttt atg   cgga gc  g
tg--  g   g    t^ ctt       -------      a-      t   agc    c  gc
         110       100               90         80        70
```

noveliR_3740dG=-68.9

```
            10        20        30        40            50         60
ga----    t                   a  ag           a---   aa-    c-|   c  g
      cgaa gcccacttccaacaacacc at  tgtccccaact    gat   aacc  caca tt a
      gctt CGGGTGAAGGTTGTTGTGG TA  ataggggttga    tta   ttgg  gtgt ag t
tgcccg    C                   C  gg           acca   atg    ac^   t  a
 130       120       110       100        90        80        70
```

noveliR_2573dG=-42.7

```
          10        20         30        40
aac  g   --|     T      T     -     Tcaaccc         a
   gg tcg  gGTTTA TGGGTT GGGT CGGGT       gacctgtta \
   ct ggc  tcgaat gcccaa ccca gccca       ctgggcaat g
ca-  g   aa^     t      -     a     -------         t
   90        80         70               60
```

noveliR_631dG=-42.8

```
            10        20        30        40        50
ta----|     a    c                   c  aa   tttta     gga
      ttatta cact caaaatctcattctacac cc  aca      tattt   a
      aataAT GTGA GTTTTTAGAGTAAGAtgtg gg  tgt      ataaa   a
cttcac^     C    A                   a  ag   tcac-     aag
 110       100        90        80        70         60
```

noveliR_3714dG=-23.8

```
                  10          20         30        40
aac---------     t-    -  -      g   -|   cag    aacc      tag
            tcttg  gct tc agcat ggt gcct   catg    cttcag   \
            AGGGC  TGA GG TCGTG Cta tgga   gtat    gaagtt   g
tatgaaatagTT     TT    T  T      G   g^   a--    a---      cct
 100        90        80        70           60            50
```

noveliR_315dG=-17.7

```
            10        20        30        40
--      ct--|  a     tc    c  a    tttttct   t
  cgtggt    gca gtct  tgg at tctg       atc t
  gtatca    CGT CGGG  ACC TG agat       tag c
tt      aaAC^  G     TC    C  A    -------   t
        70        60        50
```

noveliR_3544dG=-22.1

```
        10         20        30        40
ttttagca      C     -|    G  CCAaaat   gacc
        ctGTGT CCAC TGGGC TG       gtt    \
        gacgcg ggtg atccg ac       caa    c
atgagcca      c     c^    a  cat----   aaat
.        70        60        50
```

noveliR_4379dG=-26.8

```
          10        20         30        40
a--|     ag     TG       GTA   C-    cg  gaa
   agcggg  cCTTG  CACTGG   CGA  GACT  at   \
   tcgtct  gggat  gtgatc   gct  ctga  tg   c
ctg^     a-     ta       gaa   ac    at  taa
  80          70        60        50
```

noveliR_4038dG=-51.4

```
          10        20        30        40        50            60
--      t       aa     a      ca                  at----|       agaga
  ctccta tttgtgc  ttac gttaag  atgtcaacattttatattg       ttttttat     t
  gaggat aaACACG  AGTG CAATTC  TGCagttgtaaaatataat       aaaaaata     a
gg      -       CC     C      AG                  gataac^       gaata
 130        120        110       100        90        80        70
```

noveliR_4582dG=-40.5

```
        10          20          30           40         50
gat  tg a  GG-       C-          G      c--|    accgc       tt
   gg  a tA   GATGGG  AACGGTTAT GCggg   aggta     ggttgt  a
   cc  t at   ttgccc  ttgccaata cgtcc   ttcgt     ccaata  c
at-  gt a  aga       at          -      ata^    attta       tc
 110       100        90         80        70         60
```

noveliR_924dG=-19.4

```
        10         20             30
ttgaagttgt    atca-   --     ---|   c   ca
          tggc     gtc  gccat   ggct cta  a
          accg     CAG  CGGTA   TCGA GAT  g
ct--------    gttcC   AA     GCC^   A   tc
          70         60        50        40
```

noveliR_2852dG=-12.8

```
       10        20          30
tct  t   t   a  ag      --|   c   atac
   ct tta aga ta  ggctc  gctc tca    \
   gg aat tCT AT  TTGAG  CGGG AGT     a
gt-  t   t   C  AG      AT^   T   aaat
  70        60        50        40
```

noveliR_918dG=-19

```
       10        20        30
cag|  caa             att    a    tc
   ttc   aaatatcgcgata   ttgg aata  g
   aag   tttaTAGTGCTAT   AGCT TTAT  c
---^  cag             AAT    A    tg
   70        60        50        40
```

noveliR_1315dG=-13

```
        10        20        30
tgatt    -| T     T  A  GCTC      aaga
     gatg gA GATTG CC CT    GCGCGT    a
     ttac ct ttaat gg ga    tgtgta    t
ttt--    t^ t     -  -  actc      accc
     70        60          50        40
```

noveliR_5090dG=-19

```
        10          20        30
cacatagt    aa---|    tcc  t          gtt
        actc     attgg   ca ggtctagtg   \
        tgag     TGACC   GT TCAGGTTAC   a
t-------    cccaa^    TAA  C          Agg
       70        60        50        40
```

noveliR_3822dG=-50.6

```
             10        20        30        40
------| tt      a           ctaa           act
      gg  ggcca agggctaaaac    ggccatctcca   g
      cc  CCGGT TCCCGATTTTG    TCGGTagaggt   a
ctagaa^ tc      C           C---           ggt
  80        70        60           50
```

noveliR_4248dG=-32.1

```
           10         20        30        40        50        60
ag--  -    a   g-|    gagc          ttatttta     aa   t  atttc    tac
    gc aaca cga  gtgat    tgttggacga        tccat  tag gc     ctga   g
    cg ttgT GTT  TACTA    ACAACTTGTt        aggta  att tg     gact   t
ccaa  t    C   AG^    AA--          tacaaca-     ag   t  ctgca    tca
 130      120       110         100         90        80        70
```

noveliR_57dG=-32.1

```
           10         20        30        40        50        60
ag--  -    a   g-|    gagc          ttatttta     aa   t  atttc    tac
    gc aaca cga  gtgat    tgttggacga        tccat  tag gc     ctga   g
    cg ttgT GTT  TACTA    ACAACTTGTt        aggta  att tg     gact   t
ccaa  t    C   AG^    AA--          tacaaca-     ag   t  ctgca    tca
 130      120       110         100         90        80        70
```

noveliR_1460dG=-49.3

```
        10         20        30        40        50        60        70
ccccttc   T-|     A              c            tatacctattacttttcg      tat
       tgt  TGTGTG TCACGGTTAAGCCa gtcaacatttta                  ttttat   \
       aca  acacac agtgccaattcggt cagttgtaaaat                  aaaata   t
gt-----   ct^     c              a            ttaattaa----------      tct
 .            120       110       100        90                  80
```

noveliR_569dG=-14.1

```
        10        20        30            40
atcttc      G    A  T   GA      tt----|    ag
      cggaTC AACT CG AGT  CGTATT      atata  \
      gtttag ttgg gt tta  gtatag      tatat  t
ta----      g    a  c   a-      ctatct^    at
     80        70         60        50
```

noveliR_4729dG=-45.8

```
        10        20        30        40          50
gaag       a     c                      tc  aag-|      ttc
    tgttatt gtact taaaaatctcattctacacttc  ac    tgtattt   t
    acaataa cgTGA GTTTTTAGAGTAAGATGTGagg  tg    atataaa   t
----       c     A                      tt  aaag^      cct
     110       100        90        80        70        60
```

noveliR_3860dG=-20.5

```
        10         20         30
tttattatta      t-     ct   -     --|   a
          tacatg  ggtcg  ttc gtgta  aagt g
          gtgtat  CCAGC  GGG CACGT  TTCG g
ctg-------      tT     AT   T     GT^   a
         70        60        50        40
```

noveliR_1203dG=-17.4

```
                10         20        30
t--------       aa   agtg-   aga     ca---|     ga
         cttcatc  cca     atc   tgtct     ggaact  t
         gAGGTGG  GGT     TGG   acgga     ttttga  g
tacggggtt       A-   GGAGG   aa-     ttaag^     ac
       80         70        60         50        40
```

noveliR_406dG=-17.9

```
        10        20        30
ggtctgctcgtag    aa    a    aga--|   g
             gccg  ccgg cgga     catt t
             cggt  GGTC GTTT     GTGg t
ttaca--------    gG    G    GAGGG^   a
               60        50        40
```

noveliR_1025dG=-17.4

```
                10         20        30
t--------       aa   agtg-   aga     ca---|     ga
         cttcatc  cca     atc   tgtct     ggaact  t
         gAGGTGG  GGT     TGG   acgga     ttttga  g
tacggggtt       A-   GGAGG   aa-     ttaag^     ac
       80         70        60         50        40
```

noveliR_4745dG=-13.2

```
        10        20         30           40
gttggttttt|   A     AT    T-   A  taca---  a   cg
          GTTT TGCAG  GGAT  TGG gc       tc tca  \
          cgga acgtc  ctta  gcc cg       ag agt  a
----------^   -     --    ct   -  ttattag  g   at
          80           70        60        50
```

noveliR_269dG=-9

```
        10        20        30
cttcaaagct|     ACA     CC     Ca-   gc
          TCATTT   GAGCT  AACTT   aaa  t
          ggtaaa   ttcga  ttgaa   ttt  t
tactt-----^     c--     tc     aca   ac
 70               60        50        40
```

noveliR_2647dG=-10.4

```
        10        20         30        40
agaag    t     t       ct  -|   atttgtttc
     aaat attca tatattt  ta ctct         g
     ttta TGGGT ATGTGGG  AT GAGA         t
ttggg    t     T       TT  C^   Gtgtgtggt
  80        70        60        50
```

noveliR_2737dG=-11.4

```
         10          20         30        40
 ta    aata      --       -|   attttctctttatt  t
   aaga    aagtat  tcacatt ttaa              ct c
   tttt    tTCATG  AGTGTAG AGTT              ga a
 g-    gaag      TC       T^   GTTtttgtgtttat  a
   90        80        70        60        50
```

noveliR_1067dG=-14.1

```
         10        20        30            40
 atcttc      G    A  T   GA      tt----|    ag
       cggaTC AACT CG AGT  CGTATT      atata  \
       gtttag ttgg gt tta  gtatag      tatat  t
 ta----      g    a  c   a-      ctatct^    at
      80        70         60        50
```

noveliR_5107dG=-22

```
              10         20         30
----     c      gg      -    -|  aa    at   t
    ataga gctgtt  ctgga gact tca  ttca  cca g
    tgttt TGGCGG  GGCTT TTGG GGT  aagt  ggt t
caga     c      A-      G    C^  A-    cg   a
 80        70         60         50         40
```

noveliR_4794dG=-22.8

```
            10        20         30         40
tatta   c----|    CT   G   AT-          ga  tc
     gag     cTACA  GAT CAG   TGGGCTggt  tc  \
     ctc     ggtgt  ttg gtc   atctggccg  ag  a
gg---   tttat^    t-   -   acc          tc  ta
80            70           60         50
```

noveliR_2173dG=-18.6

```
        10        20        30        40        50
gactctac| C   G    AGC     AG  tcat  acgcttattc     aca
        ga AGG TGTG   TCTTG  CT    gg          aggat   a
        ct tcc acac   agaac  ga    cc          tccta   c
ca------^ -   m    aca     ca  ct--  ccaaytac--     aac
 .                 90        80          70          60
```

noveliR_18dG=-32.1

```
            10         20        30        40        50        60
ag--  -    a   g-|     gagc          ttatttta    aa   t  atttc    tac
    gc aaca cga  gtgat    tgttggacga         tccat  tag gc     ctga   g
    cg ttgT GTT  TACTA    ACAACTTGTt         aggta  att tg     gact   t
ccaa  t    C   AG^     AA--          tacaaca-    ag   t  ctgca    tca
 130       120       110         100         90        80        70
```

noveliR_1892dG=-42.6

```
        10        20        30        40        50         60
aattcg    t     a                   ta    gttt     a-|  gttg     gttca
      tgaa cgtgt tgttcattgtatattgtg  gtta    tcatt  ggt    tttgt     a
      actt ytaca GCAAGTAGCATGTAGCAC  CAat    agtaa  tta    aaata     t
------    t     A                   GC    attt     aa^  aaaa     aattt
       130       120       110       100        90        80        70
```

noveliR_1270dG=-33.1

```
            10        20        30        40        50
----|       ac          a   w           aa  tttc       gaa
    tgttattg  actttaaaaa ttt ttttacattcc  ac    tatattt   a
    acaataAT  TGAGGTTTTT GAG AAGAtgtgagg  tg    atataaa   a
cttc^       CA          A   T           ag  tta-       aag
 110       100        90        80        70          60
```

noveliR_3337dG=-29.3

```
              10        20        30          40         50
-----      -   tg    t  c    cc     t  --  gg    cc  -    t    --|  aa
     tttttta aat  tctc ag tatc  actaa cc  ag  gct  ac caa gaca  gca  \
     aaaaaT TTA  GGAG TC ATGG  TGGTt gg  tc  tga  tg gtt ctgt  tgt  t
ataaa      C   GT    -  -    TT     c  at  aa    at  t    -    tt^  ga
 120       110         100        90        80        70          60
```

noveliR_3761dG=-19.3

```
        10        20        30        40
atgattcga|      C   G   GTT   Gtag       gc
         tTTCTTC TTG TTG   CAG    ggcttca  \
         gaagagg gat gac   gtc    tcggggt  t
acctta---^      t   g   ---   agcg       ag
80           70           60        50
```

noveliR_5095dG=-25.8

```
               10         20          30        40         50
-------   g   g    a-   gt    -   -        ctac  t-|    tt   a
       gat aac ttgc  tgg  ttat aag aagttgag    tc  ttata  gcc a
       ctg TTG GACG  ACT  GGTA TTC ttcaactc    ag  ggtat  tgg t
gttcacc   -   -    AG   AT    C   T        ccca  tt^    tt   t
 110         100        90        80        70        60
```

noveliR_3944dG=-15.6

```
        10        20        30
attttgtatca|          g    tt  a  aaaa
           ttttgttaaag gaac  ta cg    \
           aaaACAATTTC CTTG  AT GC    g
acaccaa----^          A    TC  G  CCAc
  70            60        50        40
```

noveliR_4647dG=-34.8

```
             10         20        30         40
g----  tg-|   c      -  TT  GA       ca  t  -    t
     gc   gagg GATGCT CG  GC  GGCTGCT  gc ag aggc \
     cg   ctcc ttatgg gc  cg  tcggcgg  cg tc ttcg a
atgaa  tta^   -      t  t-  gc       aa  -  g    t
90        80         70         60         50
```

noveliR_3809dG=-27.1

```
        10        20        30        40        50        60
ct|    aaa   g    t  aa   t  a   g       gca   gggttaatatt  g
  tgtgg   ttt gttg gg  gaa ca tgc tgggagt   tcg           ag a
  atacc   aAA TAAT TC  CTT GT ATG GCCctcg   agc           tc a
tt^    aa-   G    T  A-   -  C   -       aaa   aatt-------  a
 110        100         90          80        70
```

noveliR_4442dG=-40

```
        10        20        30        40
ttgtt|  ag   C     TTAG                gcatccaccc
     aac  GTT ACCCG    CGACCCGACCcgttaa          \
     ttg  caa tgggc    gctgggttgggcaatt          a
-----^  gg   t     tgg-                ataaacataa
       90        80         70        60
```

noveliR_4982dG=-19.9

```
            10           20        30
cgac----      ---|  GGA       T     CCttt  c
        ggcagg   CTT   TTTGACT AGACT     at t
        ccgtcc   ggg   gagctgg tttga     ta a
tagtaata      tgt^  aa-       -     cgtat  g
80        70        60          50        40
```

noveliR_5035dG=-19.9

```
            10           20        30
cgac----      ---|  GGA       T     CCttt  c
        ggcagg   CTT   TTTGACT AGACT     at t
        ccgtcc   ggg   gagctgg tttga     ta a
tagtaata      tgt^  aa-       -     cgtat  g
80        70        60          50        40
```

noveliR_2122dG=-38

```
         10        20        30         40
 taa     a        C            -| aag   gga
    gattg cATAACTC TCACTTTGGTCC Ct   att   \
    ctaac gtattgag agtgaaaccagg ga   taa   a
 ---     c        r            t^ aga   ata
    80        70        60        50
```

noveliR_1177dG=-19.4

```
        10         20        30        40
ttttaaca      C    -|   CG  CCAaaat   gacc
        ctGTGT CCAC TGGG  TG       gtt    \
        gacgcg ggtg atcc  ac       caa    c
atgagccg      c    c^   aa  cat----   aaat
.        70        60        50
```

noveliR_2552dG=-11.7

```
           10         20         30        40
ttca-    t--   a   -| at     -     aa   tttctg   tg
     tttc   ttg acc ca  tagat gtctt  aca      att  \
     aaag   AAC TGG GT  ATCTA TAGGA  tgt      taa  g
gttaa    tTC   G   A^ --     G     a-   ttta--   tc
  90        80          70        60           50
```

noveliR_1598dG=-17.3

```
          10         20          30
a-  acaa-   -|      GGCC    --   cta
  ct     gaaG TCAGGAT    GAGT  GGt   \
  ga     cttc ggtcttg    ctca  ccg   a
gg  gaaag   t^      aa--    ga   cgg
       60        50          40
```

noveliR_3277dG=-21.9

```
        10        20        30        40
gacgaa|  a     AC   GCA  CC     ac  g    gg
      gag TTGAT  CTT   CG  GGCCa  tc cagt  t
      ctc ggctg  gaa   gc  ccggt  ag gtcg  t
ccg---^  a     cc   a--  t-     aa  -    gt
80           70           60         50
```

noveliR_3889dG=-23.9

```
             10         20         30        40        50
ac----   -   aa-|       tt  t   c  -     c         tattatttt
      atg gcc   cgatcata  tc gac gt tgggt tttttttat         t
      tac TGG   GCTAGTGT  AG TTG Ca attca aaaaaagtg         a
aaattt   a   CAG^       CT  -   C  g     -         ttttttatt
 110       100        90         80         70        60
```

noveliR_1999dG=-24.6

```
             10        20         30        40
---         --     G TT C    -| C     ctgacga    ga
   tttcgcaaa  gCTGA T  G GTGT GA GGGTG       ggct  \
   ygagtgttt  tggct a  t tata ct ctcac       tcga  g
gaa         gg     a gg c    g^ t     ag-----    aa
 90        80        70        60             50
```

noveliR_3367dG=-14.1

```
         10        20          30
tc-|   ttat   a  ag     --    c   atac
   catt    aga ta  ggctc  gctc tca    \
   gtaa    tCT GT  TTGAG  CGAG AGT    a
gta^   tt--   C  AG     AT    T   aagt
 70          60        50        40
```

noveliR_4137dG=-26.7

```
        10         20         30        40        50        60
atgatcga     T  C   -     -|      Tgg   tta     a      gat  t
        caAAT GA TTG ATAGA GCACCAG   gca   aaaca gctgaa   ac t
        gttta tt aat tattt tgtggtt   tgt   tttgt tgattt   tg a
--------     t  t   g     g^      ta-   ttg     a      att  a
         110       100        90         80        70
```

noveliR_21dG=-35.9

```
              10         20        30         40
ca------|    ca   GT   - C      G             -  ta
        ccttt  gGT  CGG C AGCACA CTCGATTcggagt tc  \
        ygaaa  cta  gtt g ttgtgt gggctgggtctta ag  a
gttgatta^    --   tg   t a      g             c  at
  90          80        70        60        50
```

noveliR_228dG=-12

```
        10        20         30          40
cata     a  T   GA        G-   CC    a--| a   att
    gttgc CG TTT  TGAGTTAA  GGA  Aata   tc caa   t
    caatg gt aaa  atttagtt  cct  ttat   ag gtt   t
ac--     a  t   --        aa   ca    caa^ a   gat
    90        80          70        60        50
```

noveliR_672dG=-23.9

```
             10         20         30        40        50
ac----   -   aa-|       tt  t   c  -     c         tattatttt
     atg gcc    cgatcata  tc gac gt tgggt tttttttat         t
     tac TGG    GCTAGTGT  AG TTG Ca attca aaaaaagtg         a
aaattt   a   CAG^       CT  -   C  g     -         ttttttatt
 110       100        90         80         70        60
```

noveliR_1404dG=-18.8

```
           10          20         30
ttcaa--    -       gt--     -|g   gttg
       ttca tgtatgg    cgaag t gcg    t
       aagT ACGTACC    GTTTC G Cgt    t
tgtttcg    C       ATAT     T^G   aggt
 70        60        50        40
```

noveliR_1418dG=-25.6

```
        10          20        30        40        50
ttt      ct  --  g        a         atcacct   t   tg  -  a--| gg
   agcaag  ag  tt taaactct aaaatctta       aga ttg  tg gt   cc  \
   ttgttt  tc  GG ATTTGGGA TTTTGGAat       ttt aac  gc ca   gg  t
t--      t-  TT  G        -         gtttctt   c   gt  g  aaa^ ag
.          110       100         90        80        70
```

noveliR_2994dG=-28.5

```
                10         20         30        40        50
----       ----  a-               -|        t      ataaaaataa
    ttttgtg    tg  taagccacgttaata tttatatta tatttt          \
    aaaataC    GC  ATTTGGTGCAGTTGt aaatataat ataaag          t
gaca       CAGT  CA               a^        t      aaaaacaaaa
 110       100        90        80        70        60
```

noveliR_4650dG=-49

```
            10        20        30        40         50
----        aa                     a    tcayaag-|      ttc
    tattattg  actccaaaaatctcattctac ctcc        tgtattt   t
    ataataAC  TGAGGTTTTTAGAGTAAGAtg gagg        atataaa   t
ctta        CG                     y    tttgaaag^      cct
 110       100        90        80        70        60
```

noveliR_4503dG=-58.5

```
            10         20         30        40        50
 ---    c            -     G -|            g  a     cattttatattc
    cttc catccCCTTCTG TTTGT T GGTTACGGTtaag ca gtcaa            t
    gaag gtaggggaggat aaaca a ccagtgccaattc gt cagtt            t
 tga    a            a     g c^            g  g     atttttcattta
  120       110       100        90        80        70        60
```

noveliR_1442dG=-17

```
        10        20        30         40
catatagccgACC| A    GT      A     t-    ttg
             CC CTTA  GGGATA GGCTt  gatg   t
             gg gaat  ttttat ccgga  ttat   t
a------------^ -    ma      -     tc    ttg
               70        60          50
```

noveliR_3205dG=-14.5

```
           10        20        30        40
a---| g  t     ga ta    ca     g     ttag  t      a
    aa gg aaagt  a  gtac  tgttt acttt    tg aaaaat \
    tt ct tTTCG  T  CATG  ACAAG TGAAA    gc tttttg t
aaaa^ g  -     GG GC    --     -     tt--  -      g
  90         80          70         60            50
```

noveliR_2654dG=-45.8

```
        10            20        30        40        50        60
catgt     --  g   ta--|     a         ttgtta   tgt    c   a   ctgtt
     gctta  tc ggt    cctgata cgacccgat      tcg   cgac cga cac     \
     cggat  gg CCG    GGACTGT GCTGGGCTA      ggc   gctg gct gtg     a
-----     cc  a   TTAA^     -         ttg---   tgt    t   -   cttta
      120       110       100         90           80         70
```

noveliR_1353dG=-40.2

```
             10          20        30        40        50         60
tcc----      - -   -|         gccaactc        a  tcga    gga    -   tt
       ttcggt g aat gcggggcgt         gtcagccg gc    ccga   gtaa att  \
       gagCCA C TTA CGTTCTGCG         cagtcggc cg    ggtt   cgtt tag  g
aagaaaa      G G   G^         TCTT----        g  ta--    g--    g   tt
 120       110       100            90        80            70
```

noveliR_4529dG=-33.8

```
           10        20          30        40        50
--- tg   aat        a  --|   t   g   ctt    tt  a    g     at
   a  ttc   ggttttgt ct  tacc agt gtc   tccg  ac ggac taggg  \
   t  gag   CCGGGATA GG  GTGG TTG cag   gggc  tg cctg gtctt  a
ttg gt   gT-        C  TT^   T   G   c--    cc  -    -     aa
 110       100        90        80          70          60
```

noveliR_1507dG=-23.1

```
              10        20          30        40
t-----     ac-|    CCC   G   --    C aa    tgac    aaaa
      ttagc   ttAGT   ACT GGC  GTGC A  atgt    ctta    \
      aatcg   aatca   tga ccg  cacg t  taca    gaat    a
tgcatc     agt^    a--   g   ga    - gg    tcc-    catc
        90        80          70         60         50
```

noveliR_81dG=-24

```
         10        20        30        40        50
-|    t  g               ata    aaaa     taaaattaaac
 gtgta ct tttattgtacattgt   gtta    ttatt           a
 cacat ga agATAGCATGTAGCA   CAGT    aatag           c
g^     -  -              CGC    CAAa     tccataayaaa
 100         90        80        70        60
```

noveliR_4276dG=-14.9

```
        10        20         30         40
taaagagtaaAG    T      A  -    A-|         t      g
            TGAA AGTACC GG ATTG  CAttttagtg aaaaat \
            gctt ttgtgg cc tgat  gtgaaattgc ttttta t
aaatt-------    t      a  a    aa^         -      g
  90               80        70        60
```

noveliR_3173dG=-29

```
          10          20        30        40        50
tctta  aa--  t    --|       ttg          acg    aacaca   atcg
     gg    gc cgga  gtgcttcg   gaagttttgg   caaa      gag    \
     cc    CG GCCT  TACGGAGC   TTTCaaggcc   gttt      ctt    g
aggca  aaaC  -    TT^       ---          a--    gaaa--   gaaa
 .       100         90           80          70          60
```

noveliR_385dG=-20.5

```
        10          20        30        40
gtagt          --|  aa   tc   c   ttg     at-   t
     aacgtggttt  gca  tct  tgg att   acat   atc t
     ttgtatcaaa  CGT  GGG  ACC TAG   tgta   tag c
-----          AC^  GC   CC   C   ta-     aac   c
             80        70        60          50
```

noveliR_386dG=-20.5

```
        10          20        30        40
gtagt          --|  aa   tc   c   ttg    at-   t
     aacgtggttt  gca  tct  tgg att   acat   atc t
     ttgtatcaaa  CGT  GGG  ACC TAG   tgta   tag c
-----          AC^  GC   CC   C   ta-    aac   c
             80        70        60          50
```

noveliR_1086dG=-14.3

```
         10        20        30
a-|   atca  GT  CG     A     ttatca
  gagc    aG  TT  TGGTGT GTTGG      \
  cttg    tc  ga  gtcaca caatc      c
ag^   gcc-  tg  a-      -     tgactg
         60         50         40
```

noveliR_4732dG=-15.4

```
          10        20          30
gatcg--     G      T  C-     G-|  aa
       agagc TCGATA GT  CGAGT  Gtt  \
       tttcg ggttgt ta  gctca  cag  g
gcgtccg     -      c  aa     ga^  ag
       60         50        40
```

noveliR_152dG=-31.8

```
                    10        20        30        40
---------     tt---|      tg        tgtccc          aaa
         gtgtg     agagttt  agacttt      acattgggga   c
         tACAC     TCTCAGA  TTTGAga      tgtaaccctt   a
caaagaagt     CCTGT^      GT        t-----          aga
  90        80        70        60             50
```

noveliR_29dG=-28.4

```
                10        20        30          40         50
-------     -   tg    t  c    cc     t  --  gg   cc  -   t    --|  aa
       tttta aat  tctc ag tatc  actaa cc  ag  gct  ac caa gaca  gca  \
       aaaAT TTA  GGAG TC ATGG  TGGtt gg  tc  tga  tg gtt ctgt  tgt  t
gataaaa     C   GT    -  -    TT     c  at  aa   at  t   -    tt^  ga
 120       110        100          90        80        70         60
```

noveliR_313dG=-32.1

```
           10         20        30        40        50        60
ag--  -    a   g-|    gagc          ttatttta     aa   t  atttc    tac
    gc aaca cga  gtgat    tgttggacga        tccat  tag gc     ctga   g
    cg ttgT GTT  TACTA    ACAACTTGTt        aggta  att tg     gact   t
ccaa  t    C   AG^    AA--          tacaaca-     ag   t  ctgca    tca
 130      120       110         100         90        80        70
```

noveliR_2955dG=-29.3

```
              10        20        30          40         50
-----      -   tg    t  c    cc     t  --  gg   cc  -   t    --|  aa
     ttttta aat  tctc ag tatc  actaa cc  ag  gct  ac caa gaca  gca  \
     aaaaaT TTA  GGAG TC ATGG  TGGTt gg  tc  tga  tg gtt ctgt  tgt  t
ataaa      C   GT    -  -    TT     c  at  aa   at  t   -    tt^  ga
 120       110         100        90        80        70         60
```

noveliR_5078dG=-23.8

```
         10        20        30        40        50
cttttaatca|    TT       A    GG       gc   ataattatg      tg
          CTTGG  GGTGGTA TAGC  Caggtt  cga         gagtac   \
          gggct  ttatcgt gttg  gtccag  gtt         ttcatg   g
gtattc----^    --       g    --       ta   ---------      tg
100              90        80          70
```

noveliR_278dG=-24.4

```
           10            20         30         40
---   cag        ----        gcac-    ta   -| agaatt  t
   ttc   aagtcgg    ggtttgtt     gtat  gct ct      ac a
   gag   tTTAGTC    TCAAACAA     catg  cga ga      tg c
acc   taa       AATA        ACTac    ga   t^ gcctat  g
 .        90         80         70         60         50
```

noveliR_2391dG=-20.5

```
         10          20        30         40
gtagt          --|  aa   tc   c   ttg    at-   t
     aacgtggttt  gca  tct  tgg att   acat   atc t
     ttgtatcaaa  CGT  GGG  ACC TAG   tgta   tag c
-----          AC^  GC   CC   C   ta-    aac   c
               80         70        60          50
```

noveliR_75dG=-32.2

```
         10          20         30         40             50         60
gcaatcttg   --      ataa-|  a   a     tacgytg      -----    ctta       a
         gcg  agtcaa      cca cca tccca       aatggt     tcca    caaaat a
         cgc  tCGGTT      GGT GGT GGGGt       ttacca     gggt    gttttg t
gatg-----   ca      GACGC^  -   -     -------      ttgat    cg--       t
.              110       100           90                80          70
```

noveliR_3564dG=-14.6

```
             10          20        30        40
taaat-----        tt-|ta   c       atc   tt   - ac
          agagtgct   g  gct gtaaaat   aag  att c  \
          TCTTACGG   T  CGA TGTTTTg   ttc  tga g  a
atttgttggc        CCT^GC   -       gga   tc   t ta
  90         80         70          60        50
```

noveliR_4726dG=-22.2

```
           10         20          30         40
ggtaa---      t      a  -    act-|       t      g
        agtgaa agtacc gg attg    ttttagtg aaaaat \
        ttACTT TCGTGG CC TGAC    gaaattgc tttttg t
ccttgaaa      T      G  A    AAGT^       -      g
  90         80         70         60          50
```

noveliR_2518dG=-22.2

```
           10        20          30        40
ggtaa---      t      a  -    act-|       t      g
        agtgaa agtacc gg attg    ttttagtg aaaaat \
        ttACTT TCGTGG CC TGAC    gaaattgc tttttg t
ccttgaaa      T      G  A    AAGT^       -      g
  90        80        70        60         50
```

noveliR_1294dG=-24.8

```
            10        20             30        40        50
taataa----           t  -----| aca    aa   gtaa    a   c     tacta
          catgccattag cc     cc   tggt  att    ctgt aaa tgaat     a
          GTATGGTGATC GG     GG   acta  taa    gacg ttt attta     a
tttgctctta           T  TTTAC^ Ga-    a-   aaa-    g   c     tataa
120       110       100        90          80         70        60
```

noveliR_2709dG=-29.6

```
         10        20        30
taat   -|   GG  C               C   a
    tcg gttC  TT GGTTTGGTTCGGTTT Tag t
    ggc taag  aa ccaagccaagccaaa atc c
gctt   t^   aa  -               -   a
  70        60         50         40
```

noveliR_2792dG=-10.2

```
        10        20         30        40
catatatgcaCG       G-|    A   AC  c     atg-   tt
            TTTTGAT  AGTTA GGG  CA tagtc    aat  \
            gaaatta  ttaat ctc  gt atcag    ttg  t
t-----------       gg^    -   --  t     gtaa   at
             80        70           60        50
```

noveliR_5099dG=-51.7

```
            10        20          30        40        50
tc----| t        CG  TT      G  --            aaccc         a
      tg aaatgGGT  GG  TATTGG TT  TGGGTtgggttc     gacccgtta \
      ac tttgccca  ct  gtaatc aa  gcccagcccaag     ctgggcaat g
ttgcct^ -        --  gg      g  tt            ccta-         t
 110        100          90        80        70         60
```

noveliR_1901dG=-37

```
        10        20        30         40
gag|  atg    cg     g   tta   -     g  g  at--  tc
   gct   ctcg  gcgag cgt   ctc ggctg ag cg    gc  \
   cgg   gagc  CGCTC GTA   GAG TCGGC Tc gc    cg  a
---^  cg-    aT     -   TCG   A     -  g  ggag  tt
    90         80         70         60        50
```

noveliR_3210dG=-55.4

```
        10        20        30        40         50        60
atta               CAC         C      wtt-   a       gcact     a-| t
    aaacacGTGGTGTAC   TTGTGTTCC gtcaca    aaa atttctt     catga  ga t
    tttgtgcaccacatg   gacacaagg cagtgt    ttt taagggg     gtact  tt g
----               ata         c      tact   a       ttatt     ag^ t
    130       120       110       100        90        80        70
```

noveliR_1124dG=-22.4

```
              10        20        30
acat---    -|  t      c  tg   a      t
       tccg ctc gactcg tg  tct gacttg a
       gggC GGG TTGGGT AT  GGG CTGggc a
ccaaatt    T^  T      T  TT   -      a
  70        60        50         40
```

noveliR_3549dG=-28.2

```
                10        20        30        40
t------|tg aa   t  t       aa      cat  tg    g  g
       g  a  tga gc caactag  gtagca   gt  cgag ct c
       t  T  ACT CG GTTGGTT  CGTTGT   cg  gttc gg t
gttgtgg^gt CG   -  -       C-      att  gt    -  c
 90        80          70         60        50
```

noveliR_5128dG=-17.9

```
             10         20            30        40            50
aaatatcga----        -    -     tc---    gt   tt   gtt----|      aa
             gttagaat aata tacca     tttt  ttg  gtt       ttaggtc  g
             CAATTTTA TTGT ATGGT     aaaa  aat  taa       aatttag  t
ttacattaaaAAG        C    C     Ctttr    rc   tt   argaatt^      gg
 120       110       100       90        80        70         60
```

noveliR_3908dG=-22.2

```
            10        20          30        40
ggtaa---      t      a  -    act-|       t      g
        agtgaa agtacc gg attg    ttttagtg aaaaat \
        ttACTT TCGTGG CC TGAC    gaaattgc tttttg t
ccttgaaa      T      G  A    AAGT^       -      g
  90        80        70        60          50
```

noveliR_2369dG=-40.2

```
              10          20        30        40        50         60
tcc----      - -   -|        gccaactc        a  tcga    gga    -   tt
       ttcggt g aat gcggggcgt        gtcagccg gc    ccga   gtaa att  \
       gagCCA C TTA CGTTCTGCG        cagtcggc cg    ggtt   cgtt tag  g
aagaaaa      G G   G^        TCTT----        g  ta--    g--    g   tt
 120       110       100            90        80            70
```

noveliR_2277dG=-22.2

```
            10        20          30        40
ggtaa---      t      a  -    act-|       t      g
        agtgaa agtacc gg attg    ttttagtg aaaaat \
        ttACTT TCGTGG CC TGAC    gaaattgc tttttg t
ccttgaaa      T      G  A    AAGT^       -      g
  90        80        70        60          50
```

noveliR_2455dG=-28.4

```
        10          20        30         40        50        60
gatctaacg   -| C    C    A  CG    -   tg   ttctaggattc   t    gac-     t
         gAG GA ATGA ACAA AC  AGCA caa  gcg           ata agcc    cccac \
         ttc ct tact tgtt tg  ttgt gtt  tgt           tgt tcgg    gggtg t
aactg----   a^ -    -    a  --    t   gt   -----------   t    aaaa     a
 120             110          100        90                    80
```

noveliR_932dG=-33.8

```
           10        20          30        40        50
--- tg   aat        a  --|   t   g   ctt    tt  a    g     at
   a  ttc   ggttttgt ct  tacc agt gtc   tccg  ac ggac taggg  \
   t  gag   CCGGGATA GG  GTGG TTG cag   gggc  tg cctg gtctt  a
ttg gt   gT-        C  TT^   T   G   c--    cc  -    -     aa
 110        100        90        80          70          60
```

noveliR_5106dG=-26.4

```
         10           20        30        40
attc   -   -   --|  TG       G     Cagtcgaat      ac
    ttg agg CCC  TGA  AGGTTTT CGGAG         ctttgt  \
    aac ttc ggg  gct  tccaaaa gcctc         gagacg  a
ga--   g   a   tc^  ca       -     cat------      cc
   90        80        70         60              50
```

noveliR_1489dG=-28.3

```
        10         20         30         40        50        60
agaaat   g -    T  T    A    -  a-|  atg      aa     tcatctt     cg
      cgc C ATCG GA GGGG TAGA cg  tct   tgtcga  tttca       caaca  t
      gcg g tagc tt cccc gttt gt  aga   acagtt  gaagt       gttgt  t
------   - t    m  t    c    c  yg^  ---      g-     ttc----     ac
             110       100        90            80            70
```

noveliR_4096dG=-12.8

```
        10        20           30
tct  t   t   a  ag     --|   c   atac
   ct tta aga ta  ggctc  gctc tca    \
   gg aat tCT AT  TTGAG  CGGG AGT    a
gt-  t   t   C  AG     AT^   T   aaat
  70        60        50        40
```

noveliR_2271dG=-28

```
        10          20        30        40        50
acagta  ttG        --|        CCCTa           a   atg
      cc   GGCTTTTC  GTTAAAACT     gtttttaaagt cat   \
      gg   ccgaaaag  taattttgg     taagagtttta gta   a
gta---  ttg        at^        tgttc           g   gca
100           90        80        70        60
```

noveliR_2582dG=-24.8

```
         10           20         30           40
tag  -    c  t     --- a   -   a       ---|   tt    a  tt
   gc caaa ct ggggt   g agc acg taaatct   tgtg  attt ca  \
   cg gttt gA CTCCA   C TTG TGC ATTTagg   gcac  taga gt  t
a--  t    t  C     GAA C   T   -       ctg^   t-    c  ta
   100        90        80         70        60
```

noveliR_976dG=-22.2

```
           10        20          30        40
ggtaa---      t      a  -    act-|       t      g
        agtgaa agtacc gg attg    ttttagtg aaaaat \
        ttACTT TCGTGG CC TGAC    gaaattgc tttttg t
ccttgaaa      T      G  A    AAGT^       -      g
  90        80        70        60         50
```

noveliR_4094dG=-49

```
            10        20        30        40         50
----        aa                     a    tcayaag-|      ttc
    tattattg  actccaaaaatctcattctac ctcc        tgtattt   t
    ataataAC  TGAGGTTTTTAGAGTAAGAtg gagg        atataaa   t
ctta        CG                     y    tttgaaag^      cct
 110       100        90        80        70        60
```

noveliR_4256dG=-34.9

```
         10        20         30
cgag  -   t  A      A -         -|    a
    gc ctt TG GAGCTC C TGGCTTCGG GTTcc \
    cg gag ac cttgag g attgaagcc caagg t
a---  a   c  g      c a         t^    c
     70        60        50        40
```

noveliR_4429dG=-35.1

```
        10        20        30        40        50
gagaaagtaaTC| G         T         gattc   gg   g   c  tttg
            AT TTGGGAGTC GGCTCTCAa     gga  gcg tgc tc    a
            ta aactctcgg ctgagggtt     tct  tgc acg ag    t
ct----------^ g         t         gt---   aa   -   -  tttt
           100        90        80            70           60
```

noveliR_1141dG=-17.5

```
              10         20        30
a------|      g   GA-   C         AT    t
       ctagtaa tGA   GGT TTAGGTTCG  TCTT c
       gatcgtt att   cca agtttaagc  ggaa c
gttaccc^      -   aca   -         --    a
  70        60          50          40
```

noveliR_2202dG=-37.8

```
                  10              20          30        40
----------        g  ----  -    c-| a    -    gtgacaaa    at
          gggcccga gt    gt ggtg ac  cc ggtc gggat        tggt  c
          CCCGGGTT CA    CA CCGT tg  gg ccgg cccta        accg  a
ggtgggagtc        G  GGAG  G    G  aa^ -    t    --------    ag
 100        90        80        70         60                50
```

noveliR_4580dG=-31.5

```
        10        20          30          40
c      ttt    tgt         --      --|     g   gtt
 gtcact   accg   ggggttgtg  caagag  actgca cga   \
 cagtgg   tggc   TTTCAGCAC  GTTTTC  tgatgt gtt   t
-      ---    TCC         TG      cg^     -   gta
90           80        70        60         50
```

noveliR_2794dG=-32.1

```
           10         20        30        40        50        60
ag--  -    a   g-|    gagc          ttatttta     aa   t  atttc    tac
    gc aaca cga gtgat     tgttggacga        tccat  tag gc     ctga   g
    cg ttgT GTT TACTA     ACAACTTGTt        aggta  att tg     gact   t
ccaa  t    C   AG^    AA--          tacaaca-     ag   t  ctgca    tca
 130       120       110         100         90        80        70
```

noveliR_4113dG=-33.1

```
        10         20        30        40        50
aat           TTGA-|       A        gatgttg      gtg
   gtttatgTTAA     CATGAGTG TGCTcatg       tcatga   \
   taagtacagtt     gtattcgt gtgagtac       agtact   a
---           tgtaa^       a        aattgta      ggt
  100        90        80        70        60
```

noveliR_3525dG=-23.9

```
        10        20        30        40
t|    ttt     cc       t    atag     ggtta     t
 gtact   gatca  tggttgg ggta    cggta     ccgaa a
 cgtgg   ttagT  ACTAATT TCAT    GTCat     ggttt a
t^    ---     CC       T    GCGG     ga---     t
 90           80        70        60           50
```

noveliR_4810dG=-49.1

```
        10         20        30        40        50        60        70
tcgttt         ta-| a           c                    ctatttcttttttg     tat
      ttctgtttt   tg tcacggttaag cgcgtcaatattttatatta             ttttat   \
      aggacaaaa   AC AGTGCCAATTC GTGCAGttataaaatataat             aaaata   t
------         cAC^ C           A                    aa-----------      ttt
       130       120       110       100        90                   80
```

noveliR_439dG=-23.7

```
        10         20          30
ggatc     aat- t    --|   t     atata
     tgtta    gc gacg  tggc gccac     \
     acggt    CG CTGT  ACCG CGGTG     t
aa---     gcaC T    AA^   T     Cacag
70           60        50        40
```

noveliR_1704dG=-29

```
             10         20          30
---     --    at  -          a    --|    aa
   tatgt  gagg  ga gagcttttgg ttgt  gagggt  g
   gtaca  TTCC  CT CTCGGGAATC GAta  ttcccg  g
taa     tt    C-  C          A    tt^    ga
80        70         60        50        40
```

noveliR_4755dG=-26.4

```
         10            20        30        40
attc   -   -   --| TG       G     Cagtcgaat     ac
    ttg agg CCC  TGA  AGGTTTT CGGAG         ctttgt  \
    aac ttc ggg  gct  tccaaaa gcctc         gagacg  a
ga--   g   a   tc^ ca       -     cat------     cc
   90        80        70         60              50
```

noveliR_1698dG=-35.5

```
            10           20        30
g----        cTG  T    ---|          G  t  ag
     ctgattag   GC GGTC   TATACCACTTG TC ct  \
     ggctgatc   cg ctag   atgtggtgaac ag gg  g
gaaag        ta-  t    taa^          g  t  ac
  80        70         60        50        40
```

noveliR_2762dG=-36.8

```
            10        20             30        40        50
taataa----           t  -----| aca    aa   gtaa    a   c     tacta
          catgccattag cc     cc   tggt  att    ctgt aaa tgaat     a
          GTATGGTGATC GG     GG   acta  taa    gacg ttt attta     a
tttgctctta           T  TTTAC^ Ga-    a-   aaa-    g   c     tataa
120       110       100        90          80         70        60
```

noveliR_3187dG=-39.2

```
        10          20           30        40        50
agct       c       --      at--  -|   tccaat   ca       tat
    ggctgtt aggactc  tctggc    tc agcg      gtg  ggctcat   c
    tcggtag tTCTGAG  GGACTG    AG tcgt      cgc  ccgggta   a
g---       t       TT      CTCT  C^   ttttc-   aa       caa
  110       100        90        80         70        60
```

noveliR_4318dG=-17.9

```
               10             20        30
------   a   -   -----|     c          aat
      tta aag cca     aatttt tgggctaaaa   t
      aat TTC GGT     TTGGGA ACCCGatttt   t
ctcata   a   T   AGAGG^     -          cag
  70        60        50         40
```

noveliR_1676dG=-30

```
                10        20        30
c--------|    ay   act     g  a      aattt  aca
         gtgag  ccc   tatgg tc cacgtg     cc   \
         cACTC  GGG   GTACC AG GTGCAc     gg   c
gcactaacc^    A-   CT-     G  -      caac-  cta
  80        70          60         50
```

noveliR_1593dG=-49

```
            10        20        30        40         50
----        aa                     a    tcayaag-|      ttc
    tattattg  actccaaaaatctcattctac ctcc        tgtattt   t
    ataataAC  TGAGGTTTTTAGAGTAAGAtg gagg        atataaa   t
ctta        CG                     y    tttgaaag^      cct
 110       100        90        80        70        60
```

noveliR_1958dG=-49

```
            10        20        30        40         50
----        aa                     a    tcayaag-|      ttc
    tattattg  actccaaaaatctcattctac ctcc        tgtattt   t
    ataataAC  TGAGGTTTTTAGAGTAAGAtg gagg        atataaa   t
ctta        CG                     y    tttgaaag^      cct
 110       100        90        80        70        60
```

noveliR_1363dG=-20.1

```
        10        20        30        40
ttgaaaaa|    CCACC      G  Aaaact        c
        tgGTC     GAGCGT CC      atgttctt t
        actag     tttgca gg      tataagga t
ca------^    tcc--      g  gtgacc        g
             70          60        50
```

noveliR_4508dG=-20.2

```
        10        20        30
aataaac| gT      TA    AG    TCa   tat
       ga  CCGTTG  GTCT  TTGG   gga   t
       ct  ggcggc  caga  agcc   tct   c
aa-----^ tg      c-    a-    cac   cgm
70             60          50        40
```

noveliR_5026dG=-14.1

```
        10        20        30            40
atcttc      G    A  T   GA      tt----|    ag
      cggaTC AACT CG AGT  CGTATT       atata  \
      gtttag ttgg gt tta  gtatag       tatat  t
ta----      g    a  c   a-      ctatct^    at
     80        70         60        50
```

noveliR_1888dG=-24.7

```
           10        20        30            40
att      ---        a  a    ga   aa  at----|    agg
   ggaagc   gactaagg ac attt  gac  cc       ccttg   \
   tctttg   TTGGTTCC TG TAGG  TTG  Gg       ggaac   t
g--      gag        G  A    --   AG  aaaatt^    ctt
   90        80        70          60        50
```

noveliR_2399dG=-57.1

```
        10        20        30          40        50
  ttc  c     T    C                -|   y       ay
gg   ct tcCGG TCCT TTTGTGAGGATTctgg gatc ctttatg  c
cc   ga aggcc agga aaacactcctagggcc ctag gaaatac  g
  tat  t     t    t                t^   t       ag
 .        90        80        70        60
```

noveliR_4834dG=-23.7

```
                 10          20         30
---------   a         a--       ag   c-   ttg-|    g
         gca cagaaaagg   gccaaa  cat  att    gaagc g
         tGT GTTTTTTTC   CGGTTT  gta  tag    cttcg t
cgaggacct   G         GTA       ag   ca   ttta^    g
90        80        70        60        50        40
```

noveliR_2304dG=-32.2

```
        10          20         30        40             50        60
gcaatcttg   --      ataa-|  a   a     tacgytg      -----    ctta      a
         gcg  agtcaa       cca cca tccca      aatggt     tcca    caaaat a
         cgc  tCGGTT       GGT GGT GGGGt      ttacca     gggt    gttttg t
gatg-----   ca      GACGC^  -   -     -------      ttgat    cg--      t
.            110       100          90                80          70
```
